# The effects of Guozhuang dance on exercise self-efficacy in coronary heart disease patients following percutaneous coronary intervention: a randomized controlled trial

**DOI:** 10.3389/fcvm.2025.1688894

**Published:** 2026-01-19

**Authors:** Wenxiao Wu, Fangqun Mao, Yijiang Zhou, Jing Wang, Xia Qian, Xiting Huang, Yang Liu, Yong Fang

**Affiliations:** 1 School of Nursing, Zhejiang Industry Polytechnic College, Shaoxing, Zhejiang, China; 2 Department of Nursing, the First Affiliated Hospital, Zhejiang University School of Medicine, Hangzhou, Zhejiang, China; 3 Department of Nursing, the Fourth Affiliated Hospital of School of Medicine, and International School of Medicine, International Institutes of Medicine, Zhejiang University, Jinhua, Zhejiang, China; 4 School of Nursing, Changsha Medical University, Changsha, Hunan, China

**Keywords:** Guozhuang dance, percutaneous coronary intervention, exercise self-efficacy, 6-minute walk test, quality of life

## Abstract

**Background:**

Coronary heart disease (CHD) is a leading cause of death. Despite PCI improving coronary perfusion, many patients face low physical activity, reduced self-efficacy, and poor quality of life, emphasizing the need for better rehabilitation.

**Objectives:**

To evaluate the effects of a 12-week Guozhuang dance intervention on exercise self-efficacy, functional capacity, physical activity, and health-related quality of life in CHD patients following PCI.

**Methods:**

88 post-PCI patients were randomly assigned to a control group (*n* = 44) or an intervention group (*n* = 44). Both groups received standard care; the control group participated in conventional rehabilitation, while the intervention group added Guozhuang dance. Outcomes measures were assessed at baseline(T0), at 4(T1) and 12(T2) weeks of intervention, and at 6 weeks after post-intervention (T3) using the Multidimensional Self-Efficacy for Exercise Scale (MSES), Six-Minute walk distance (6MWD), International Physical Activity Questionnaire (IPAQ), and the Chinese Questionnaire of Quality of Life (CQQC). Data were analyzed using intention-to-treat (ITT) analysis. The protocol was registered in the Chinese Clinical Trial Registry (ChiCTR2300069341).

**Results:**

Both groups demonstrated comparable baseline characteristics (*P* > 0.05). Significant group × time interactions were found for exercise self-efficacy and 6MWT distance (both *P* < 0.001), while physical activity and quality of life showed significant main effects of group (both *P* < 0.05). The intervention group demonstrated significantly greater improvements in exercise self-efficacy, physical activity, and quality of life at T1, T2, and T3 (all *P* < 0.05). For 6MWD, significant between-group differences were observed at T2 and T3 (both *P* < 0.001), but not at T1 (*P* = 0.065 > 0.05).

**Conclusion:**

A 12-week Guozhuang dance program significantly enhances exercise self-efficacy, functional capacity, physical activity, and quality of life in CHD patients after PCI, making it a valuable addition to traditional cardiac rehabilitation.

**Clinical Trial Registration:**

https://www.chictr.org.cn/bin/home, identifier, ChiCTR2300069341.

## 1 Introduction

Coronary Heart Disease (CHD) is the leading cause of mortality among patients with cardiovascular conditions (1, 2). Percutaneous coronary intervention (PCI) is a primary treatment approach for CHD (2); however, patients remain at risk of restenosis in the coronary arteries, including at the stent site, following the procedure (3). Post-PCI prognosis is closely associated with the effectiveness of cardiac rehabilitation (4), however, patient participation and adherence to these programmes remain suboptimal, with 60.8% of patients failing to attain the physical activity levels recommended by clinical guidelines—underscoring a critical gap between prescribed rehabilitation and actual patient engagement (5). This gap is often due to factors such as lack of motivation, insufficient social support, and unengaging intervention content (6). This shortfall reflects low rehabilitation adherence, and exercise self-efficacy has been identified as one of the most important determinants of cardiac rehabilitation adherence (7). Exercise self-efficacy (ESE)—defined as an individual's belief in their ability to perform and adhere to physical activity—is a critical factor in successful rehabilitation (8). Higher ESE levels are linked to greater adherence to exercise regimens. Unfortunately, most patients post-PCI exhibit low ESE, lack confidence in maintaining regular physical activity, and consequently have reduced activity levels, which may contribute to recurrence of disease, physical disability, or even mortality (7, 9). Existing interventions have failed to effectively improve ESE, highlighting the need for more engaging and sustainable rehabilitation strategies. Guozhuang dance is a comprehensive aerobic activity rooted in Tibetan cultural traditions, originating from communal singing and dancing around a hearth, it was inscribed on the first list of National Intangible Cultural Heritage of China approved by the State Council (10). Its characteristic movements emphasize “force”, “speed”, and “range”, which help participants experience physical satisfaction and may adherence enhance exercise self-efficacy (11). The subjective perceived exertion of Guozhuang dance ranges from 11 to 14 on the Borg scale (12), with a maximum heart rate (HRmax) of 120–150 bpm, aligning with the American Heart Association's recommendations for post-PCI exercise rehabilitation in CHD patients (13, 14). This study aims to evaluate the feasibility and effectiveness of Guozhuang dance as a culturally tailored intervention to improve ESE among CHD patients following PCI. By fostering greater confidence and sustained physical activity engagement, this approach has the potential to address critical gaps in cardiac rehabilitation and improve long-term cardiovascular outcomes.

## 2 Methods

### 2.1 Participants

This 12-week, single-center, randomized controlled trial was conducted at the First Affiliated Hospital of Zhejiang University School of Medicine in Hangzhou, Zhejiang Province, China, from February to September 2023. Participants completed face-to-face assessments at four time points: on the day of discharge (baseline), at 4 weeks into the intervention, at 12 weeks (end of intervention), and at 18 weeks (6-week follow-up post-intervention). The protocol was approved by the hospital's Ethics Committee (Approval No. 20230048-K) and registered in the Chinese Clinical Trial Registry (ChiCTR2300069341); all procedures adhered to the Declaration of Helsinki. All participants provided written informed consent before enrolment.

Sample size (*n*) was calculated using: *n* = 2*σ*^2^(*t_α_ *+ *t_β_*)^2^/(*μ*_1_–*μ*_2_)^2^, with two-sided *α* = 0.05, power (1 − *β*) = 0.80, and 1:1 allocation (15). Pilot data yielded mean post-intervention exercise self-efficacy scores of 46.33 ± 2.73 (intervention) vs. 39.83 ± 13.89 (control), giving 39 participants per arm (Supplementary 2). Allowing for 15% attrition, we enrolled 44 per group (total *N* = 88). Between February and September 2023, 88 coronary heart disease patients post-PCI were randomized 1:1 via a computer-generated Excel sequence by staff independent of the study team. Wards A and B—equivalent in patient diagnoses, treatment protocols, and staffing—were designated as the intervention and control arms, respectively, to minimize contamination and allocation bias. The inclusion criteria were (1) Met the diagnostic criteria for stable coronary heart disease according to the 2018 Chinese Guidelines, (2) Aged 18–75 years, (3) Undergoing PCI for the first time, (4) Had 1–2 stents implanted, (5) Classified as Class II or III in cardiac function according to the New York Heart Association (NYHA) classification, (6) Hemodynamically stable, mentally alert, able to communicate, and willing to participate in the study. Exclusion Criteria were (1) Patients with severe heart failure, malignant tumors, or other conditions affecting walking or respiratory training, (2) Individuals with mental, personality, cognitive, or consciousness disorders, (3) Patients with limited mobility or those unsuitable for follow-up.

### 2.2 Intervention

#### 2.2.1 Control group

Both groups received standard post-PCI care (Supplementary 1), including: Upon discharge, all patients received standard post-PCI care, including a secondary prevention handbook covering medication management, dietary guidance, wound care, and follow-up schedules. Patients were advised to perform at least 150 min of moderate-intensity aerobic exercise per week, with options including walking, Tai Chi, Baduanjin, calisthenics, jogging, or swimming. Exercise activities were self-recorded using examples provided (e.g., “walking 30 min/day, 5 days/week”).

#### 2.2.2 Intervention group

In addition to standard post-PCI care, patients in the intervention group received the Guozhuang dance intervention on top of the control group's measures (10, 16) (Supplementary 1). Prior to the intervention, all team members underwent standardized training led by the lead supervisor and were required to pass a competency assessment. Pre-discharge interventions included the following: ① Guozhuang Dance Instruction: On the day of discharge, implementation team taught the patients the Guozhuang dance movements. Instruction sessions were video-recorded for reference and feedback. Patients were guided to perform the movements correctly, and were required to independently complete at least one full cycle of the Guozhuang dance routine before discharge (Supplementary 3). ② Pre-exercise Counseling: A specialist nurse instructed patients using the ‘Secondary Prevention Knowledge for Coronary Heart Disease after PCI’ handbook (Supplementary 4) and explained the benefits and precautions of physical activity. Patients were also taught to self-monitor their heart rate and were advised to perform the exercise 0.5–1.0 h after meals, with exercise intensity to be increased gradually. Post-discharge interventions included the following: ① Exercise Execution: Patients followed the FITT (Frequency, Intensity, Time, Type) principle to carry out Guozhuang dance-based cardiac rehabilitation. Frequency: Three times per week. Duration: 12 weeks of intervention, approximately 45 min per session. Intensity: Targeted at 50%–60% of the patient's estimated maximum heart rate (220−age) × 0.5 ∼ 0.6, or a perceived level of light sweating and warmth (6). Respiratory rate should increase by 40%–50% from baseline resting levels (17). Type: Aerobic exercise—Guozhuang dance. ② Exercise Monitoring: Patients received reminders via the “Xiao Daka” WeChat mini-program and telephone calls from the research team. They were instructed to log their activity each week and complete a coronary rehabilitation diary. Patients were supported and encouraged regularly. Weekly exercise totaling over 150 min was considered compliant; patients failing to meet this standard for two consecutive weeks were excluded from the study. ③ Feedback and Communication: A dedicated WeChat group named “Guozhuang Dance” was created to facilitate peer interaction and problem-solving. Patients were encouraged to share their experiences, raise questions, and seek guidance from the research team. The platform provided a space for collaborative learning, feedback exchange, and discussion of the intervention process.

### 2.3 Outcomes

#### 2.3.1 Multidimensional self-efficacy for exercise scale (MSES)

The Multidimensional Self-Efficacy for Exercise Scale (MSES), revised by Dong Jianxiu in 2022 (18), was validated for reliability and validity specifically in patients with coronary heart disease. The scale comprises three dimensions: Items 1–3 assess “Task Self-Efficacy”, “Items 4–6 assess”, “Coping Self-Efficacy”, and “Items 7–9 assess Scheduling Self-Efficacy”. The Cronbach's *α* coefficient for the scale was 0.92, and the test-retest reliability coefficient was 0.86, indicating high internal consistency and good reliability.

#### 2.3.2 Six-minute walk distance (6MWD)

The 6 min walk distance (6MWD) test is a widely used, simple, and safe method for evaluating patients’ aerobic endurance and is commonly applied in cardiac rehabilitation assessments (19). The procedure involves setting a 30 m straight corridor in the ward with a quiet, interruption-free environment. Patients are encouraged to walk back and forth as fast as possible within 6 min. Vital signs are closely monitored during the test, and any symptoms such as angina or dyspnea prompt immediate cessation of the activity or rest in place.

#### 2.3.3 International physical activity questionnaire (IPAQ)

The International Physical Activity Questionnaire (IPAQ) has been applied in physical activity assessments across 51 countries (20). The questionnaire consists of six sections: (1) Work-related physical activity (2) Transportation (3) Housework (4) Sports and leisure activities (5) Sitting time (6) Sleep duration Physical activity levels are quantified using Metabolic Equivalent of Task (MET), where 1 MET corresponds to an oxygen uptake of 3.5 mL/kg/min. Based on IPAQ scoring standards, activity levels are classified as low, moderate, or high.

#### 2.3.4 Chinese questionnaire of quality of life in patients with cardiovascular diseases (CQQC)

The CQQC, developed by Jiangsheng Liu et al. (21), evaluates quality of life across six dimensions: physical activity, disease status, medical care, general life, psychosocial aspects, and work. The total score ranges from 0 to 154, with lower scores indicating greater limitations and higher scores reflecting fewer limitations and a better quality of life. Notably, the extreme values of 0 and 154 do not imply complete health or death. The content validity index of the scale is 0.90, and the Cronbach's *α* coefficient is 0.92, indicating strong reliability and validity.

### 2.4 Data collection and quality control

Data were collected at four predefined time points: baseline (T0), 4 weeks into the intervention (T1), 12 weeks at intervention completion (T2), and 18 weeks (T3, 6 weeks post-intervention). At T0, participants completed the General Information Questionnaire, the MSES, IPAQ, CQQC, and their 6MWD was measured. At T1, T2, and T3, both groups repeated. During each survey, investigators used standardized instructions to explain the purpose and significance of the study and guided patients through the questionnaire process. They checked the completeness of the responses on-site to minimize missing data.

To ensure objectivity, investigators were blinded to group assignments. Two designated researchers were responsible for follow-up data management: one collected and organized the data, while the other verified and corrected it, ensuring the accuracy and integrity of the dataset.

### 2.5 Statistical analysis

All analyses were conducted based on the intention-to-treat (ITT) principle, including all randomized participants in their assigned groups regardless of protocol adherence or study completion. A separate per-protocol (PP) analysis was conducted comparing only the 81 completers (intervention *n* = 40; control *n* = 41), and additional models adjusting for baseline covariates were used to assess the robustness of primary findings (Supplementary 5). Data were analyzed using SPSS version 26.0. The normality of the distribution was tested using the Kolmogorov–Smirnov test, the Levene test was used to check the homogeneity of variance, and sphericity with the Mauchly test. A descriptive analysis of the sociodemographic variables was performed. Data is presented as mean (standard deviation) when the variables were quantitative and presented normal distribution; otherwise, data is presented as median (interquartile range). For the nominal variables, frequencies were obtained and are presented in counts (percentages). Given the nature of repeated measurements and potential autocorrelation and missing values across time points for the same patient, a Generalized Estimating Equations (GEE) model (22) was employed for statistical analysis. Baseline data were treated as covariates to adjust the results accordingly. A significance level of *α* = 0.05 was adopted, with *P* < 0.05 considered statistically significant.

## 3 Results

### 3.1 General characteristics of the study subjects in the two groups

#### 3.1.1 Comparison of general characteristics between the two groups

A total of 88 patients were enrolled in this study, with 44 patients in the intervention group and 44 in the control group. The mean age of patients in the intervention group was (62.59 ± 6.70) years, while that in the control group was (61.52 ± 8.15) years. Among them, 29 patients (65.91%) in the intervention group and 33 patients (75.00%) in the control group were male. Regarding occupational status, 52.27% of participants were retired or on medical leave, while 15.91%, 9.10%, and 22.72% were unemployed, part-time employed, and fully employed, respectively. In the intervention group, 22.73% of patients reported exercising 3–7 times per week, whereas 40.91% reported almost no physical activity. Furthermore, 56.82% of patients exercised for less than 30 min per session, and only 4.54% exercised for more than 60 min per session. Concerning post-PCI stent implantation, 67.05% of patients received a single stent. There were no statistically significant differences in baseline demographic or clinical characteristics between the two groups (*P* > 0.05), indicating comparability and balance. Detailed information is presented in Table 1.

**Table 1:** Comparison of general characteristics between two groups.

Characteristics	Intervention group (*n* = 44)	Control group (*n* = 44)	Value	*P*
	Mean (SD)/Median (range)/*n*%			
Age (years, x̄ ± s)	62.59 ± 6.70	61.52 ± 8.15	0.671^a^	0.504
Gender			0.873^b^	0.350
Male	29 (65.91)	33 (75.00)		
Female	15 (34.09)	11 (25.00)		
Education level			2.377^c^	0.526
Primary school or below	14 (31.82)	20 (45.45)		
Junior high school	17 (38.64)	12 (27.27)		
High school	11 (25.00)	9 (20.45)		
College or above	2 (4.54)	3 (6.83)		
Marital status			0.000^c^	1.000
Married	40 (90.90)	39 (88.64)		
Single	4 (9.10)	5 (11.36)		
Current work status			3.025^b^	0.388
Unemployed	7 (15.91)	7 (15.91)		
Retired or sick	23 (52.27)	21 (47.73)		
Part-time	4 (9.10)	1 (2.27)		
Full-time	10 (22.72)	15 (34.09)		
Household monthly income (CNY)			1.492^c^	0.474
<2,000	1 (2.27)	0 (0)		
2,000–4,000	12 (27.27)	11 (25.00)		
>4,000	31 (70.46)	33 (75.00)		
Living arrangement			4.598^b^	0.100
Nursing home	0 (0)	2 (4.55)		
Living with spouse	34 (77.27)	37 (84.09)		
Living with children/friends	10 (22.73)	5 (11.36)		
Medical insurance type			3.567^c^	0.168
Full out-of-pocket	20 (45.45)	18 (40.91)		
Insurance reimbursement	23 (52.27)	21 (47.73)		
Rural cooperative	1 (2.27)	5 (11.36)		
Post-PCI time			0.723^c^	0.395
≤1 year	38 (86.36%)	35 (79.55%)		
>1 year	6 (13.64%)	9 (20.45%)		
Family history of CHD			0.180^b^	0.914
Yes	3 (6.82)	4 (9.09)		
No	41 (93.18%)	40 (90.91%)		
Exercise types			0.088^b^	0.767
One type	14 (31.82)	13 (29.55)		
Two or more types	27 (61.36)	27 (61.36)		
Exercise frequency			2.668^c^	0.102
Frequent (2–3/week)	10 (22.73)	15 (34.09)		
Occasional (1–2/month)	16 (36.36%)	16 (40.91%)		
Rare or none (<2/month)	18 (40.91%)	11 (25.00%)		
Exercise duration			1.396^c^	0.514
<30 min/session	25 (56.82)	30 (68.18)		
30–60 min/session	17 (38.64)	13 (29.55)		
>60 min/session	2 (4.54)	1 (2.27)		
Angiographic report			−1.550^c^	0.121
Single-vessel disease	21 (47.73)	13 (29.55)		
Double-vessel disease	20 (45.45)	28 (63.63)		
Triple-vessel or above	3 (6.82)	3 (6.82)		
Number of stents			1.286^b^	0.257
One	32 (72.73)	27 (61.36)		
Two	12 (27.27)	17 (38.64)		

^a^ Independent *t*-test.

^b^ Chi square test.

^c^ Mann–Whitney *U* test.

#### 3.1.2 Participant attrition

**Figure 1:**
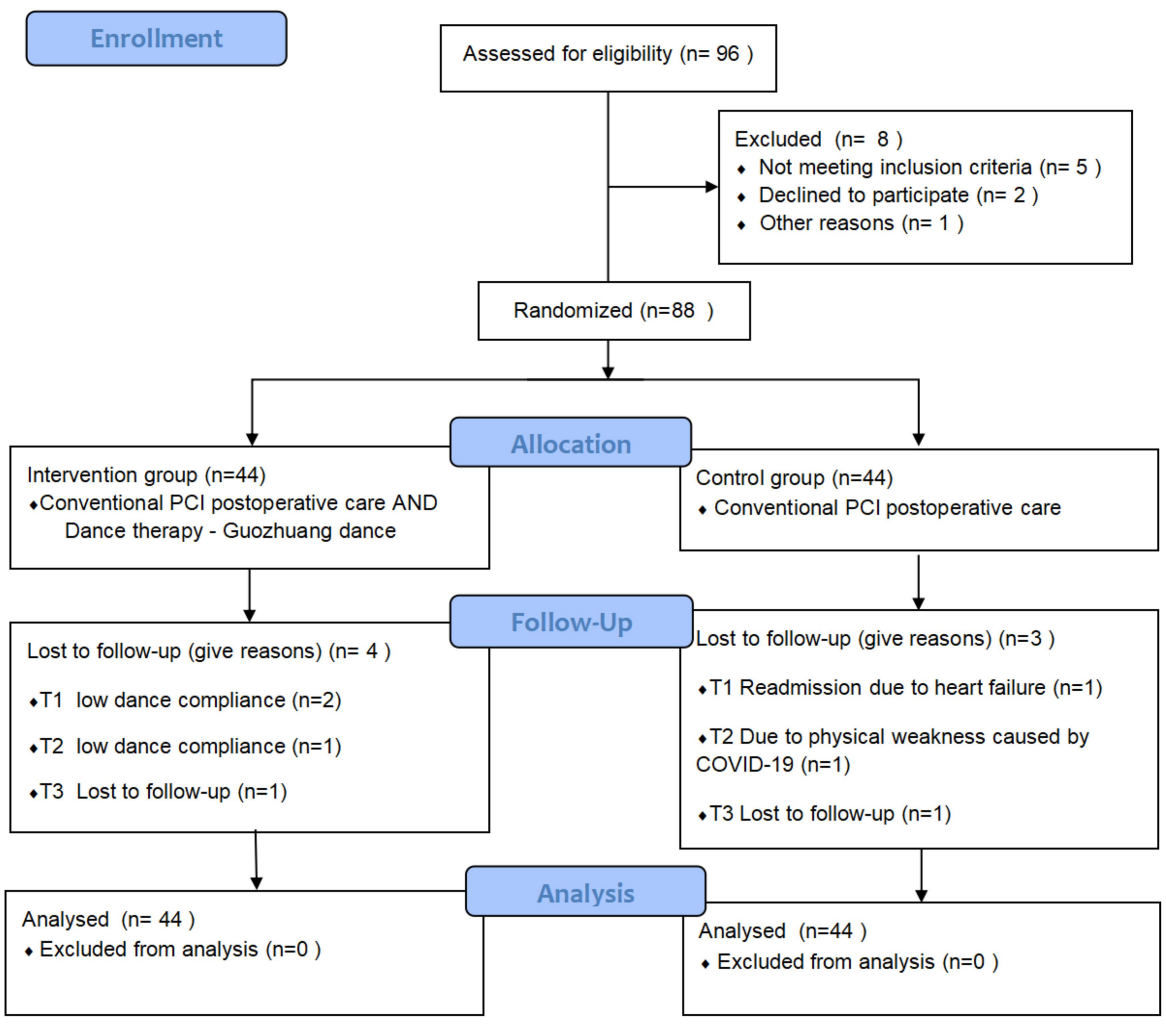
Participant attrition flowchart. This flowchart illustrates the participant recruitment and attrition process throughout the study. A total of 88 patients were randomized into two groups: the intervention group (*n* = 44) and the control group (*n* = 44). Attrition due to inability to complete the exercise protocol and loss to follow-up is depicted, with a final total of 81 participants completing the study (40 in the intervention group and 41 in the control group).

During the course of the study, five participants withdrew due to their inability to complete the exercise protocol—three from the intervention group and two from the control group. Additionally, two participants were lost to follow-up, one from each group. Details are presented in Figure 1. A total of 81 participants completed the study, including 40 in the intervention group and 41 in the control group, resulting in an overall attrition rate of 7.95%.

### 3.2 Scale scores at each time point in both groups

**Figure 2:**
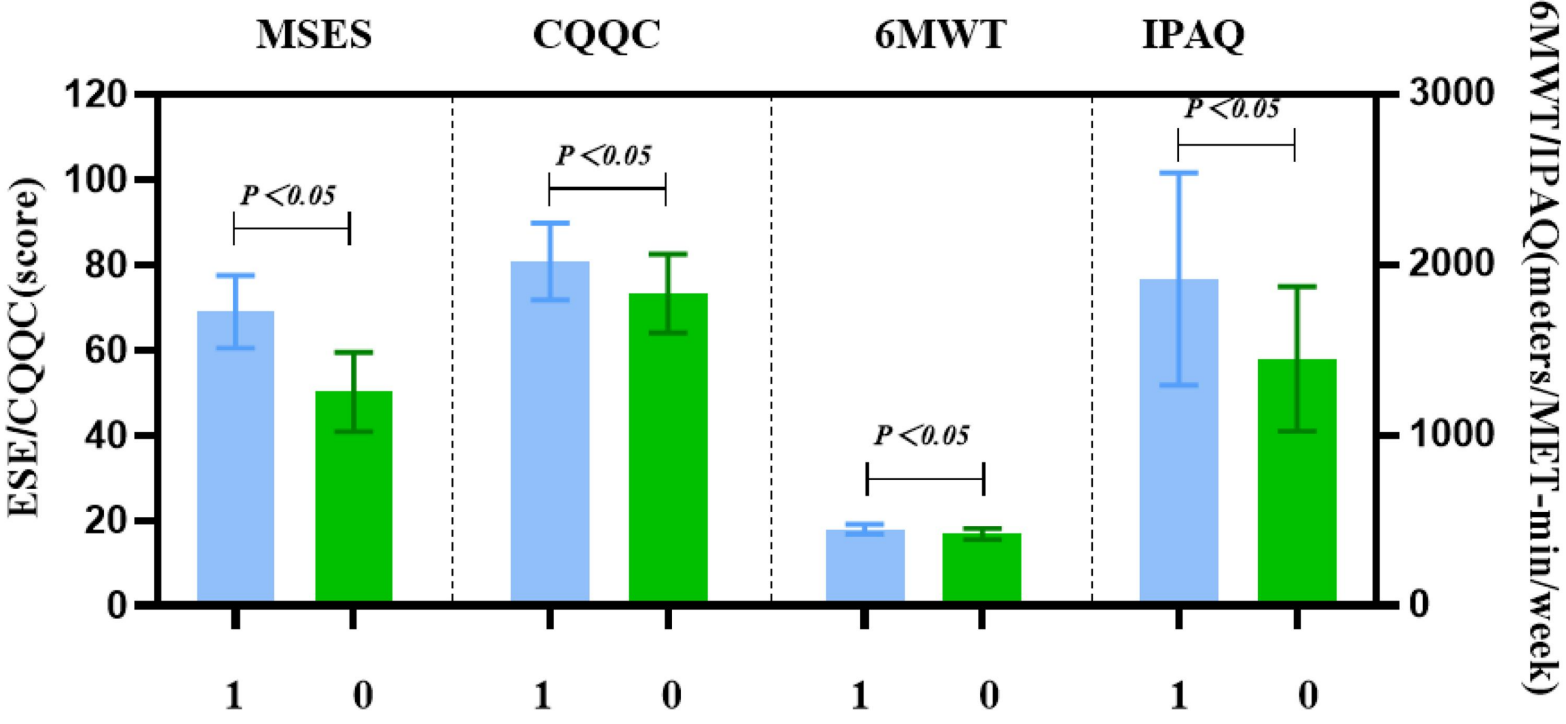
Bar chart of scale scores at week 12 for both groups. This bar chart presents the mean scores for exercise self-efficacy (MSES), quality of life (CQQC), 6-minute walk distance (6MWD), and physical activity (IPAQ) at week 12 (T2) for both the intervention and control groups. The intervention group showed significantly higher scores across all measures compared to the control group, highlighting the effectiveness of the Guozhuang dance intervention.

The scale scores at each time point for both groups are summarized in Table 2. After 12 weeks of intervention, the intervention group had a significantly higher MSES score (67.84 ± 9.76) compared to the control group (50.90 ± 9.12), with a statistically significant difference (*P* < 0.05). The quality of life score in the intervention group was (80.23 ± 8.96), higher than that in the control group (73.77 ± 9.13), also showing a statistically significant difference (*P* < 0.05). The 6MWD was (447.57 ± 30.42) meters in the intervention group and (422.05 ± 33.58) meters in the control group, with a statistically significant difference between the two groups (*P* < 0.05). The physical activity score was 1,837.00 (1,619.17, 2,115.25) points in the intervention group and 1,365.50 (1,120.25, 1,625.75) points in the control group, with a statistically significant difference between the two groups (*P* < 0.05). After the 12-week intervention, statistically significant differences were observed in scale scores between the two groups, as shown in Figure 2.

**Table 2:** Scale scores of patients in both groups at each time point.

Time	Outcomes	MSES	CQQC	6MWD	IPAQ
T0	Intervention group	37.70 ± 9.99	57.30 ± 8.72	402.86 ± 42.26	598.50 (431.25, 785.00)
	Control group	36.93 ± 10.09	55.59 ± 9.37	411.16 ± 39.21	577.50 (443.00, 720.00)
	*t/Z*	0.522	1.177	−0.613	0.830
	*p*	0.605	0.240	0.542	0.409
T1	Intervention group	55.32 ± 10.79	70.34 ± 8.87	437.82 ± 36.03	1,430.50 (1,190.50, 2,020.50)
	Control group	44.91 ± 9.18	65.46 ± 7.96	423.52 ± 35.66	1,123.00 (891.75, 1,369.36)
	*t/Z*	4.536	2.946	1.870	3.513
	*p*	<0.001	0.003	0.065	<0.001
T2	Intervention group	67.84 ± 9.76	80.23 ± 8.96	447.57 ± 30.42	1,837.00 (1,619.17, 2,115.25)
	Control group	50.90 ± 9.12	73.77 ± 9.13	422.05 ± 33.58	1,365.50 (1,120.25, 1,625.75)
	*t/Z*	6.280	3.505	3.755	4.214
	*p*	<0.001	<0.001	<0.001	<0.001
T3	Intervention group	72.81 ± 8.79	86.15 ± 6.64	448.04 ± 28.48	1,787.64 (1,561.50, 2,112.00)
	Control group	54.03 ± 8.86	79.65 ± 7.13	426.11 ± 30.36	1,360.50 (1,163.25, 1,627.76)
	*t/Z*	6.789	4.415	3.697	4.219
	*p*	<0.001	<0.001	<0.001	<0.001

T0, baseline; T1, 4 weeks into the intervention; T2, 12 weeks (end of intervention); T3, 18 weeks (6-week follow-up post-intervention). MSES, multidimensional self-efficacy for exercise scale; CQQC, Chinese questionnaire of quality of life in patients with cardiovascular diseases; 6MWD, six-minute walk distance; IPAQ, international physical activity questionnaire.

### 3.3 Comparison of exercise self-efficacy scores between the two groups

#### 3.3.1 Comparison of exercise self-efficacy scores at different time points

**Figure 3:**
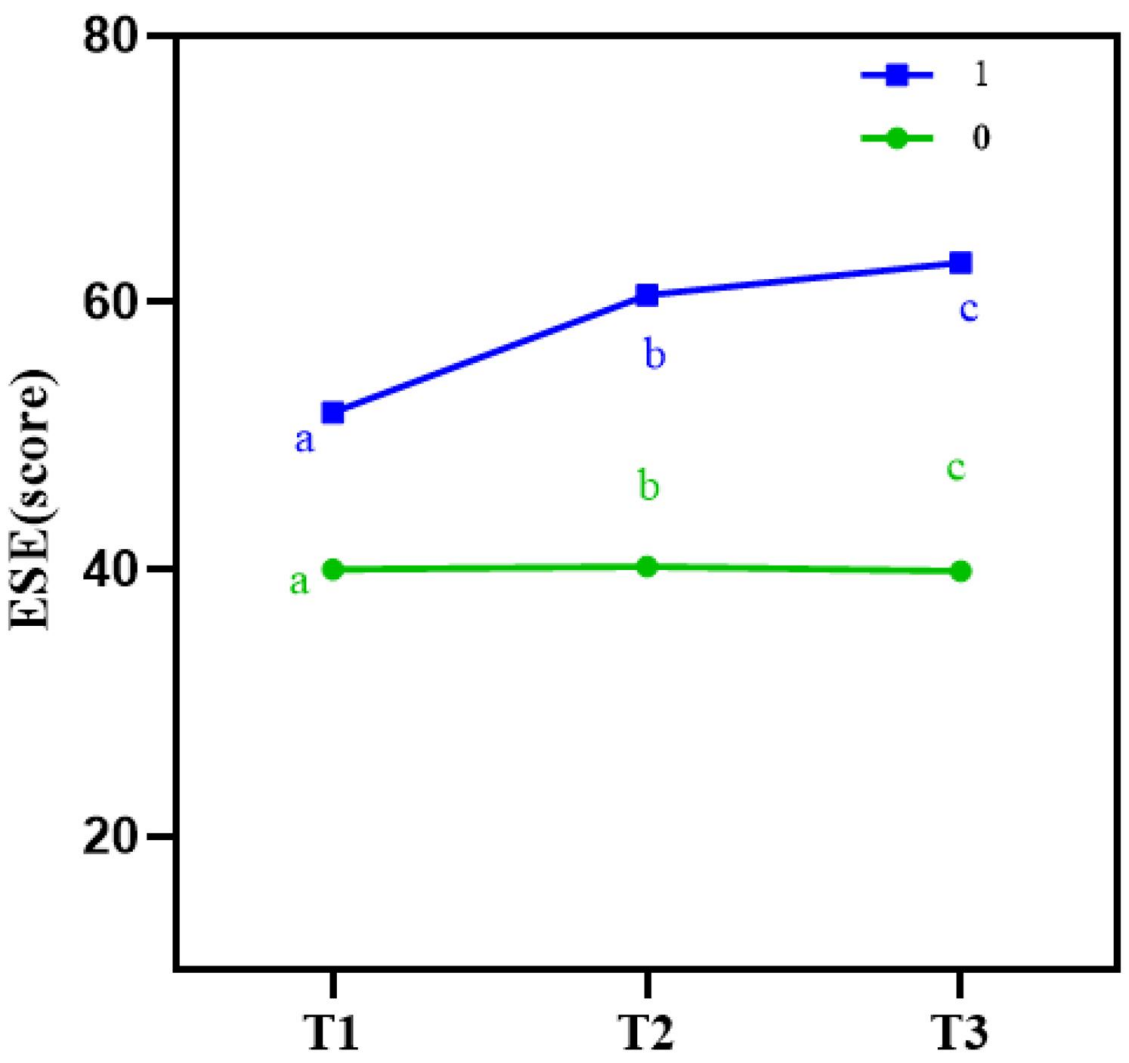
Line chart of exercise self-efficacy (ESE) scores at different time points in both groups. This line chart tracks the changes in exercise self-efficacy (ESE) scores over the study period for both the intervention and control groups. The intervention group showed a significant and consistent increase in ESE scores at 4 weeks (T1), 12 weeks (T2), and 6 weeks post-intervention (T3), while the control group exhibited no significant changes over time.

(a) To explore the effect of the intervention on exercise self-efficacy (ESE) in both groups, a generalized estimating equation (GEE) was used to analyze the repeated measures data. The results showed a significant interaction effect between time and group for the MSES score (*P* < 0.001), as shown in Table 3. This indicates that Guozhuang dance had differential effects on MSES scores across different time points. The trend of score changes over time is illustrated in Figure 3. Further analysis of the main effects of time and group is warranted.

**Table 3:** Model effect estimation using generalized estimated equation (*n* = 88).

Outcome	Group		Time		Group*time	
	Wald *χ*^2^ (df)	*P*	Wald *χ*^2^ (df)	*P*	Wald *χ*^2^ (df)	*P*
MSES	325.53 (1)	<0.001	196.44 (2)	<0.001	173.84 (2)	<0.001
6MWD	72.47 (1)	<0.001	13.80 (2)	0.001	18.75 (2)	<0.001
IPAQ	12.68 (1)	<0.001	8.24 (2)	0.016	2.96 (2)	0.228
CQQC	22.90 (1)	<0.001	664.05 (2)	<0.001	3.69 (2)	0.158

MSES, multidimensional self-efficacy for exercise scale; 6MWD, six-minute walk distance; IPAQ, international physical activity questionnaire; CQQC, Chinese questionnaire of quality of life in patients with cardiovascular diseases; df, degrees of freedom.

(b) **Main effect of time**: Using T0 as a covariate to adjust for baseline effects, the results showed that, in the control group, MSES scores at T2 and T3 did not differ significantly from those at T1 (*P* > 0.05). In contrast, in the intervention group, MSES scores at both T2 and T3 were significantly higher than at T1 (*P* < 0.001), as shown in Table 4.

**Table 4:** The comparison of outcome variables by generalized estimating equation.

Characteristics	MSES		6MWD		IPAQ		CQQC	
	Wald *χ*^2^ (df)	*β* (95% CI), *p*	Wald *χ*^2^ (df)	*β* (95% CI), *p*	Wald *χ*^2^ (df)	*β* (95% CI), *p*	Wald *χ*^2^ (df)	*β* (95% CI), *p*
Between groups								
IG vs. CG	46.23 (1)	−10.31 (−13.28, −7.34), <0.001	31.35 (1)	−21.14 (−28.54, −13.74), <0.001	6.91 (1)	557.97 (142.04, 973.90), <0.001	15.00 (1)	−3.80 (−5.72, −1.8), <0.001
Within groups								
IG (T1)								
IG (T2)	139.82 (1)	12.65 (10.55, 14.74), <0.001	18.62 (1)	10.65 (5.81, 15.48), <0.001	29.99 (1)	225.34 (144.68, 305.99), <0.001	210.83 (1)	9.97 (8.62, 11.31), <0.001
IG (T3)	163.54 (1)	17.82 (15.09, 20.55), <0.001	14.87 (1)	11.01 (5.41, 16.61), <0.001	21.51 (1)	224.10 (129.39, 318.81), <0.001	240.96 (1)	15.99 (14.00, 18.01), <0.001
CG (T1)								
CG (T2)	177.89 (1)	5.83 (4.97, 6.68), <0.001	1.68 (1)	−2.24 (−5.62, 1.14), 0.194	1.66 (1)	209.79 (−109.63, 529.21), 0.198	173.36 (1)	8.30 (7.06, 9.53), <0.001
CG (T3)	164.06 (1)	8.95 (4.97, 10.32), <0.001	0.87 (1)	1.88 (−2.07, 5.82), 0.351	0.23 (1)	80.19 (−244.70, 405.08), 0.629	422.83 (1)	14.51 (12.80, 15.50), <0.001

T1, 4 weeks into the intervention; T2, 12 weeks (end of intervention); T3, 18 weeks (6-week follow-up post-intervention). MSES, multidimensional self-efficacy for exercise scale; 6MWD, six-minute walk distance; IPAQ, international physical activity questionnaire; CQQC, Chinese questionnaire of quality of life in patients with cardiovascular diseases. IG, intervention group; CG, control group.

(c) **Main effect of group:** Using T0 as a covariate to adjust for baseline effects, the results showed that, at both T2 and T3, the MSES scores in the intervention group were significantly higher than those in the control group (*P* < 0.001). Furthermore, the mean difference between the two groups increased progressively over time, as shown in Table 4.

#### 3.3.2 Comparison of MSES dimensional scores between the two groups

**Figure 4:**
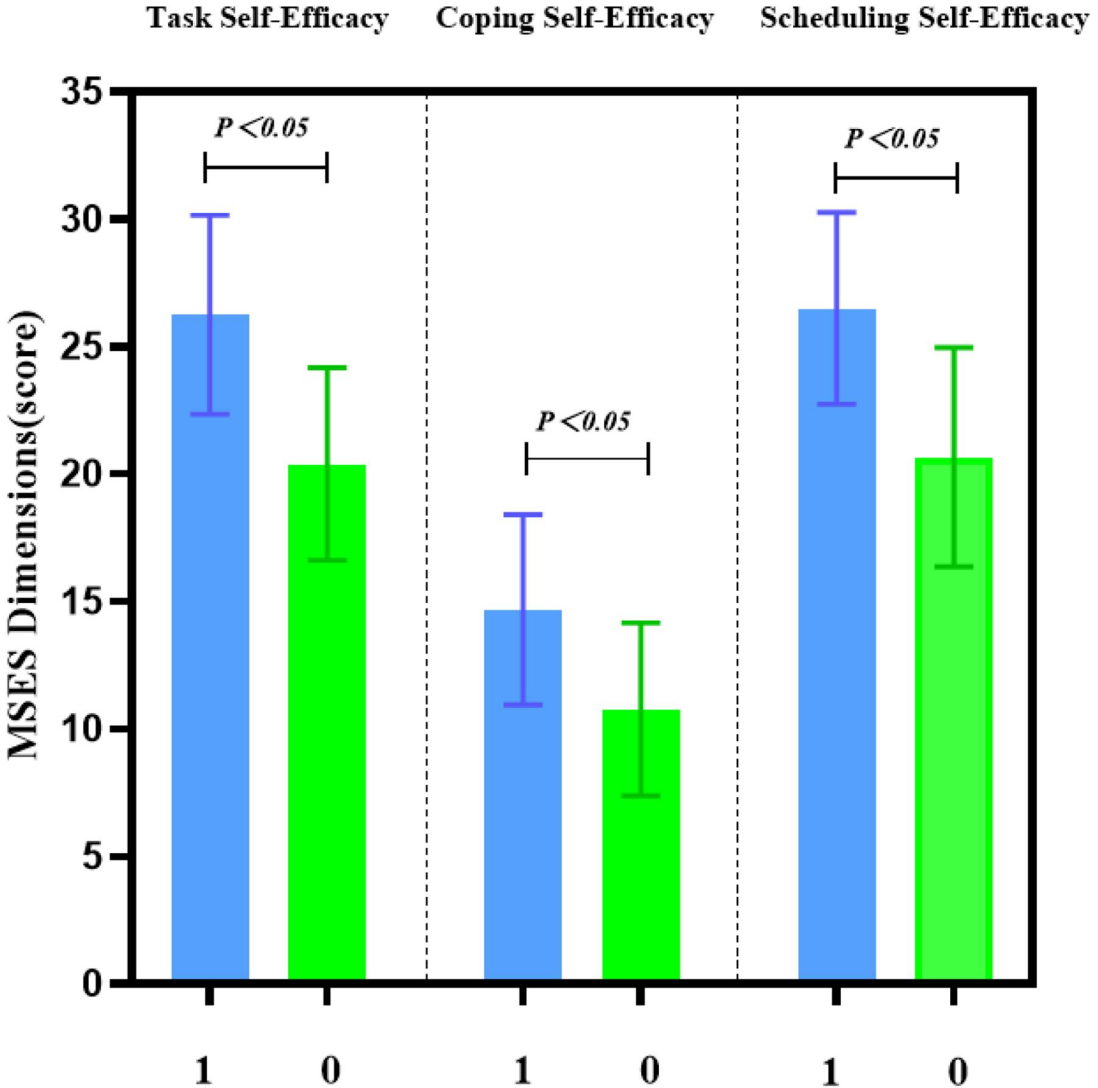
Comparison of exercise self-efficacy dimensions between the two groups. This figure compares the exercise self-efficacy dimensions (task self-efficacy, coping self-efficacy, and scheduling self-efficacy) between the intervention and control groups at week 12. The intervention group showed significantly higher scores across all three dimensions, with the greatest improvement observed in task self-efficacy, emphasizing the impact of Guozhuang dance on patients’ confidence in physical activity.

At week 12, the intervention group had a total ESE score of (67.84 ± 9.76), while the control group scored (50.90 ± 9.12). Within the intervention group, the Task Self-Efficacy score was (24.72 ± 4.63), the Coping Self-Efficacy score was (13.72 ± 3.59), and the Scheduling Self-Efficacy score was (23.89 ± 4.75). These results indicate that problem-solving efficacy was the lowest among the three dimensions in both groups, as detailed in Table 5. Statistically significant differences were observed between the two groups across all dimensions (*P* < 0.001), as illustrated in Figure 4.

**Table 5:** Dimensional scores of MSES in both groups at week 12 (mean ± SD).

MSES dimensions	Intervention group (*n* = 44)	Control group (*n* = 44)	*t*	*p*
Task Self-Efficacy	24.72 ± 4.63	17.32 ± 5.03	7.181	<0.001
Coping Self-Efficacy	13.72 ± 3.59	9.52 ± 2.88	6.044	<0.001
Scheduling Self-Efficacy	23.89 ± 4.75	17.16 ± 4.22	7.030	<0.001

MSES, multidimensional self-efficacy for exercise scale.

### 3.4 Comparison of 6 MWD at different time points between the two groups

**Figure 5:**
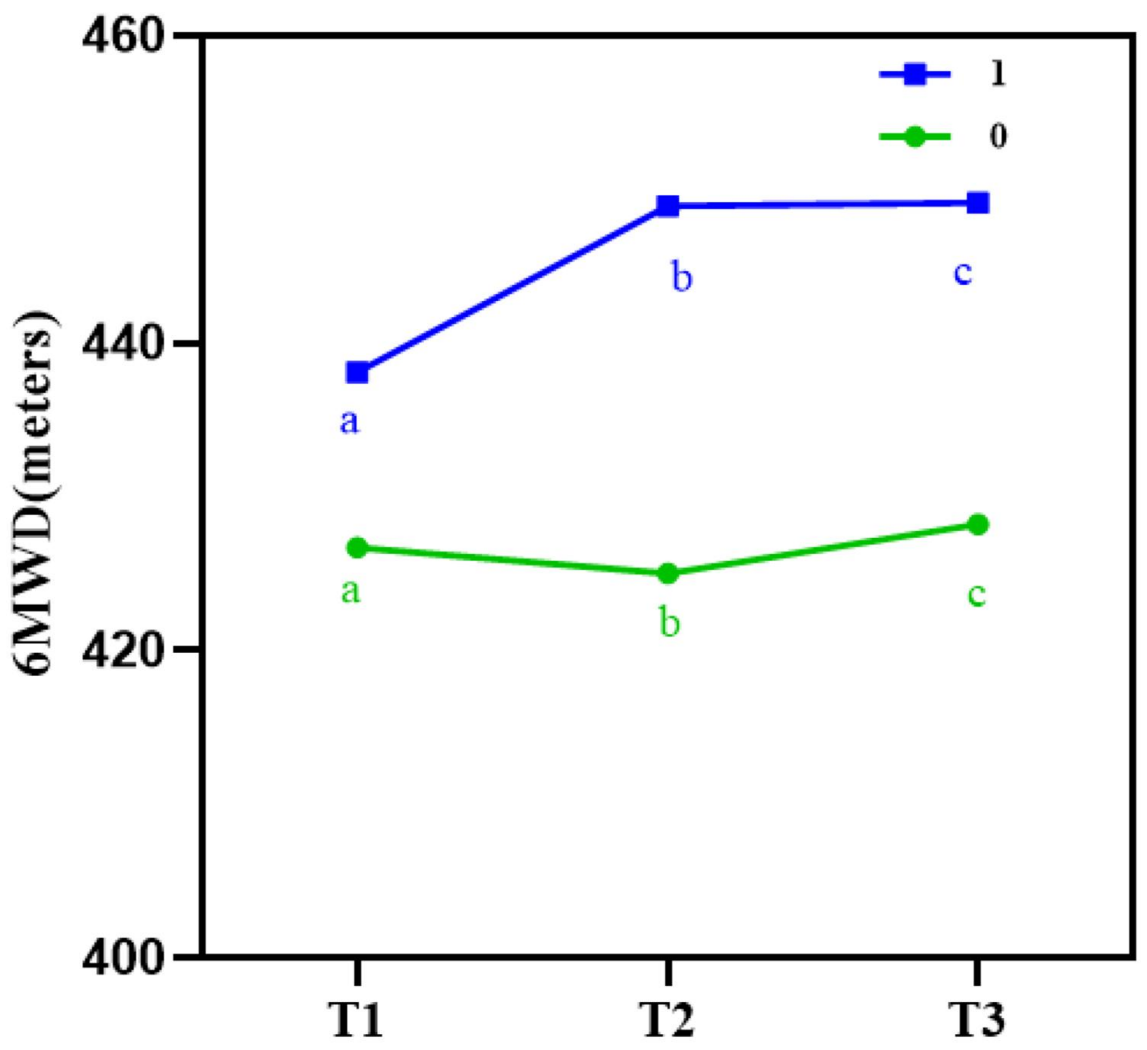
Line chart of 6-minute walk distance (6MWD) at different time points in both groups. This line chart illustrates the change in 6-minute walk distance (6MWD) scores at baseline (T0), 12 weeks (T2), and 6 weeks post-intervention (T3). The intervention group demonstrated a significant improvement in walking distance over time, while the control group showed no significant change, indicating the positive effects of Guozhuang dance on functional capacity and cardiovascular health.

(a) Generalized estimating equation analysis revealed significant differences in 6 MWD between the intervention and control groups at different time points (*P* < 0.001). A significant interaction effect was also observed between time and intervention (*P* < 0.001), as shown in Table 3. These findings suggest that Guozhuang dance had varying effects on 6MWD over time, as illustrated in Figure 5, warranting further analysis of the main effects of time and group.

(b) **Main effect of time**: Using T0 as a covariate to adjust for baseline effects, the results showed that in the control group, 6MWD at all subsequent time points did not differ significantly from T1 (*P* > 0.05). In contrast, in the intervention group, 6MWD at T2 and T3 was significantly higher than at T1 (*P* < 0.001). Moreover, the mean difference between the two groups increased progressively over time, as shown in Table 4.

(c) **Main effect of group**: Using T0 as a covariate to adjust for baseline effects, the results showed that, compared to the control group, the intervention group exhibited significantly higher 6MWD at both T2 and T3 (*P* < 0.001), as shown in Table 4.

### 3.5 Comparison of physical activity at different time points between the two groups

**Figure 6:**
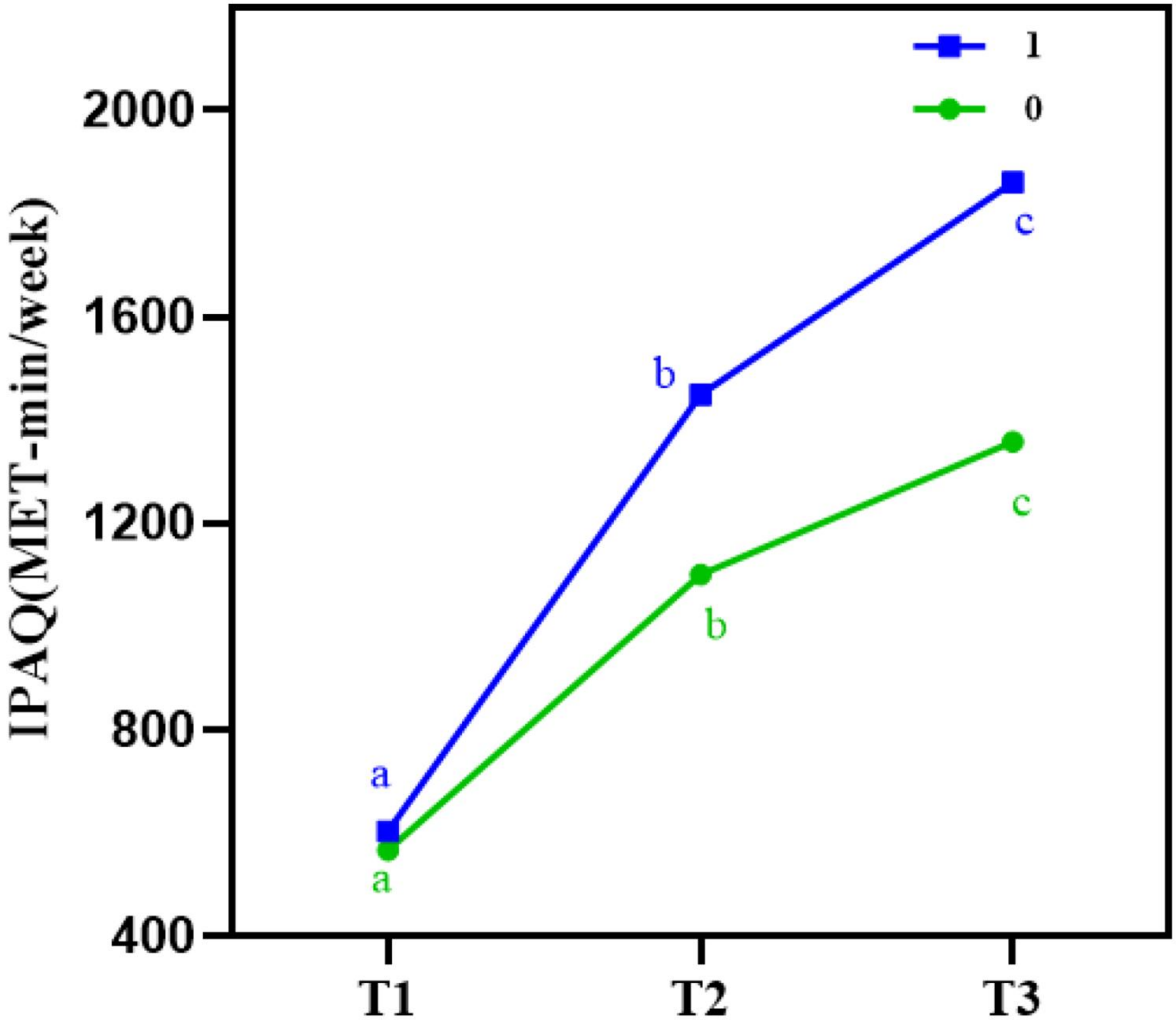
IPAQ at different time points in both groups. This figure shows the trend of physical activity levels as assessed by the International Physical Activity Questionnaire (IPAQ) at different time points (T0, T1, T2, T3). The intervention group displayed a significant increase in physical activity scores compared to the control group, reflecting the role of Guozhuang dance in promoting sustained physical activity among post-PCI patients.

(a) GEE analysis showed that IPAQ scores differed significantly between the control and intervention groups at different time points (*P* < 0.001). However, no significant interaction effect was found between time and intervention (*P* = 0.228 > 0.05), indicating a focus on the main effects. Details are presented in Table 3, and the trends in physical activity over time are illustrated in Figure 6.

(b) **Comparison of main effects:** Using T0 as a covariate to adjust for baseline effects, the results showed a significant main effect of time on physical activity scores, with a Wald *χ*^2^ value of 8.24 (*P* < 0.05). A significant main effect of group was also observed for IPAQ scores (*P* < 0.05). These findings indicate that physical activity levels in post-PCI patients varied across different time points, and that the Guozhuang dance intervention had a statistically significant effect on improving physical activity.

Using T1 as the reference point, a significant difference in physical activity was observed at T2 (*P* < 0.05). Compared to the control group, the intervention group also showed significantly higher physical activity levels (*P* < 0.05), as presented in Table 4.

### 3.6 Comparison of quality of life at different time points between the two groups

**Figure 7:**
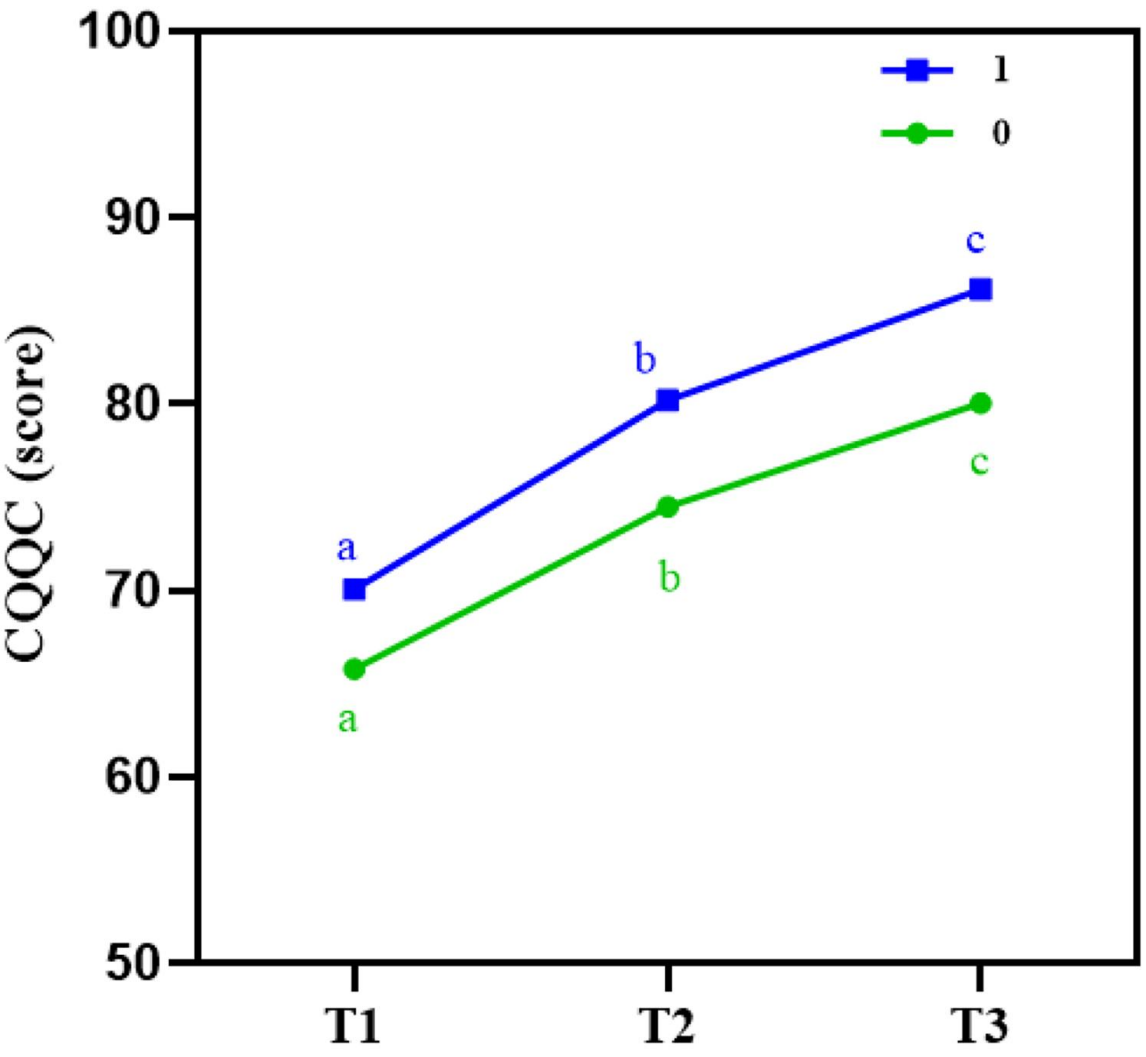
Line chart of quality of life (CQQC) at different time points in both groups. This line chart depicts the changes in quality of life scores, as measured by the Chinese Questionnaire of Quality of Life in Cardiovascular Diseases (CQQC), over the study period. The intervention group experienced a significant improvement in quality of life at 12 weeks (T2) and 6 weeks post-intervention (T3), particularly in the psychosocial and disease-related domains, compared to the control group.

(a) With the extension of the intervention period, quality of life improved in both groups. However, the degree of improvement was consistently greater in the intervention group compared to the control group across all time points, with the difference being statistically significant (*P* < 0.001). No significant interaction effect was found between time and intervention (*P* = 0.158 > 0.05), indicating that the main effects should be the focus. Detailed results are shown in Table 3, and the trend in quality of life over time is illustrated in Figure 7.

(b) **Comparison of main effects:** Using T0 as a covariate to adjust for baseline effects, the results showed a significant main effect of time on CQQC, with a Wald *χ*^2^ value of 664.05 (*P* < 0.05). A significant main effect of group was also observed (*P* < 0.05), indicating that quality of life levels in post-PCI patients differed across time points. The Guozhuang dance intervention had a statistically significant impact on quality of life improvement. Using T1 as the reference, a significant difference in quality of life was found at T2 (*P* < 0.05). Compared to the control group, the intervention group demonstrated a statistically significant improvement in quality of life (*P* < 0.05), as shown in Table 4.

## 4 Discussion

### 4.1 Guozhuang dance effectively improves ESE in post-PCI patients with CHD

Results of this study indicated that on the day of discharge, post-PCI patients exhibited a low level of ESE, consistent with the low values reported by Wong (18). After the intervention, the intervention group showed significantly higher total ESE and subscale scores at all follow-up time points compared to the control group (*P* < 0.001), mirroring the results reported by Lin Yuanzheng et al. (10, 14). Guozhuang dance appears to enhance ESE by eliciting positive emotions during exercise and bolstering confidence in physical activity (12). These effects can be explained through Bandura's four sources of self-efficacy (23): ① Mastery experiences (direct experience): The dance routines were structured to progress from simple to more challenging movements (11, 16), allowing participants to perceive their own improvement and to derive a sense of achievement. ② Vicarious experiences (indirect experience): Observing peers with similar conditions successfully complete rehabilitation tasks strengthens one's belief in achieving the same goals (24). In our study, intervention-group patients shared videos of their Guozhuang dance sessions via WeChat, discussed challenges, and exchanged coping strategies—activities that promoted engagement and further elevated their ESE. ③ Verbal persuasion: Positive encouragement from the research team—including regular monitoring of postoperative recovery, reinforcement of the importance of cardiac rehabilitation, and motivational prompts—helped patients believe in their capacity to perform the prescribed exercises (25). ④ Emotional arousal: Initial nervousness and occasional lapses in memory of the dance steps diminished over time as participants grew more confident (26). The simple, enjoyable nature of Guozhuang dance satisfied intrinsic needs for competence and enjoyment, transforming emotional states into motivational drivers that further enhanced ESE (10). Notably, both groups scored lowest on the problem-solving subscale of the Multidimensional Self-Efficacy for Exercise Scale (MSES), indicating that this dimension remains the most challenging to improve and highlighting the need for the research team to focus future interventions on helping patients identify and overcome specific exercise barriers. The difficulty in enhancing problem-solving self-efficacy may stem from the complex, individualized nature of the barriers that patients face, such as difficulties in time management, family responsibilities, or environmental constraints (27). To address this, future interventions should focus on strategies that help patients identify specific barriers to exercise and develop practical solutions. For example, time management strategies can help patients set a fixed exercise schedule or break their exercise into shorter sessions, making it easier to integrate into daily life (28). Social support strategies, such as involving family members in the exercise process or joining a group activity, can provide the encouragement and accountability needed to sustain exercise (29). Additionally, environmental adjustments such as creating a convenient and supportive exercise space at home, or introducing enjoyable and culturally relevant activities like Guozhuang dance, can remove some of the obstacles that patients face (12, 28, 30).

### 4.2 Guozhuang dance effectively improves 6 MWD in post-PCI patients with CHD

The results of this study revealed that the 6 min walk distance (6 MWD) in the intervention group was significantly greater than that in the control group following the intervention. As a sensitive indicator of clinical status changes after cardiac rehabilitation and a reliable measure of cardiac function, the 6MWD results suggest that Guozhuang Dance may enhance both cardiac function and overall physical performance. These findings are consistent with prevailing views in the literature (16, 25, 31).

This improvement may be attributed to the dance's natural and life-oriented movement patterns. Guozhuang dance incorporates gestures that mimic daily labor—such as threshing barley or spinning wool—or imitate natural elements, such as the soaring of an eagle (11, 30). These immersive, relatable movements help participants engage more fully in the activity, promoting muscle relaxation and emotional release. Regular aerobic exercise, such as Guozhuang dance, enhances heart function by improving stroke volume and reducing resting heart rate, ultimately leading to better cardiac output during physical activity (32). As a result, the heart is able to pump blood more efficiently, thereby increasing oxygen delivery to muscles during exercise, which is reflected in improved walking performance. Additionally, Guozhuang dance may improve endothelial function, which plays a critical role in vascular health. Endothelial dysfunction is common in patients with CHD, leading to reduced blood flow and impaired exercise tolerance. The rhythmic movements of Guozhuang dance may promote better nitric oxide production, enhancing vasodilation and improving blood flow to muscles and tissues during exercise. This effect can contribute to improved exercise capacity, as greater blood flow enhances muscle oxygenation and reduces fatigue (33). Another potential mechanism is the improvement in heart rate recovery. Cardiovascular fitness, developed through regular exercise, enhances the ability of the autonomic nervous system to regulate heart rate (34). Guozhuang dance, by promoting rhythmic and continuous movement, may improve the heart's ability to return to resting heart rate more quickly after exercise, which is associated with better cardiovascular health and enhanced exercise capacity (12). As a result, patients may experience improvements in cardiac function, pulmonary ventilation, and exercise tolerance, ultimately contributing to increased 6MWD.

### 4.3 Guozhuang dance effectively enhances physical activity levels in post-PCI patients with CHD

As the intervention period progressed, the trend in physical activity levels between the two groups showed significant differences over time (*P* < 0.001), consistent with findings reported by Barbara et al (35). One possible reason for this improvement is that dance therapy, by integrating music, movement, and sensory stimulation, provides a non-pharmacological intervention with both psychological and physiological benefits (36, 37). Moreover, Guozhuang Dance is not limited by space, equipment, or time. Even individuals with no prior experience can easily learn it, making it particularly feasible and effective in improving cardiopulmonary fitness and cardiovascular function among middle-aged and elderly patients (11). This accessibility increases patient participation rates, in contrast to traditional forms of physical exercise, which are often perceived as monotonous and lack variety, thus failing to maintain patient motivation (38). Importantly, ensuring patient safety and appropriate exercise intensity was a priority throughout the intervention. In addition to the paper-based rehabilitation log, a digital monitoring tool, the “Xiao Daka” WeChat mini-program, was introduced. This allowed patients to log their exercise type, duration, and other relevant details. The research team reviewed these logs daily, providing timely feedback to ensure adherence to the prescribed exercise regimen. This system not only facilitated regular monitoring but also fostered a sense of accountability among patients. Furthermore, to ensure that each patient participated in a safe and tailored exercise program, all participants underwent a rigorous exercise capacity assessment conducted by a trained rehabilitation therapist prior to initiating the Guozhuang dance sessions. This assessment was critical in determining each patient's baseline fitness level and in designing a personalized exercise regimen that started at a safe intensity and gradually increased over the course of the intervention. This individualized approach was key to minimizing the risk of overexertion while promoting gradual improvements in physical capacity. Additionally, exercise self-efficacy is a critical factor influencing physical activity levels in post-PCI patients (39). An increase in exercise self-efficacy helps to stabilize exercise behaviors. Regular encouragement, personalized feedback, and ongoing attention during the intervention process further reinforce patients’ confidence in engaging in physical activity (40), encouraging adherence and successful task completion.

### 4.4 Guozhuang dance helps improve quality of life in post-PCI patients with CHD

This study showed that Guozhuang Dance can improve quality of life in post-PCI patients over time, aligning with the findings of Mariam et al. (41) on the effects of dance therapy in patients with coronary heart disease. This may be explained by the fact that quality of life is a subjective, multidimensional, and dynamic concept involving physical, emotional, cognitive, social, and role-related aspects (42).

Previous research suggests that dance therapy may promote the generation of new neurons in the dentate gyrus and lead to positive changes in brain structure and function (43). Guozhuang Dance incorporates elements of music therapy by integrating emotionally evocative songs such as “Heavenly Road” and “Gesang Flowers on the Grassland” (11, 25), thereby creating an emotionally engaging and relaxed exercise environment. Additionally, the movements of Guozhuang Dance—modeled on human labor and animal behaviors—help relieve physical and mental tension. As a result, it is a spontaneous, enjoyable, and safe intervention modality (38).

This study found that scores in the disease-related and psychosocial dimensions of quality of life improved over time. Notably, a significant difference in overall quality of life between the two groups emerged from week 6 of the intervention (*P* < 0.001), suggesting that Guozhuang Dance may offer relatively immediate benefits. These findings are consistent with the results reported by Linda Cox et al (16). Overall, Guozhuang Dance may help patients adopt a more positive attitude toward daily life, enhance self-awareness and social functioning, and promote sustained improvements in quality of life.

### 4.5 Differential temporal patterns of intervention effects

A notable finding of this study is the distinct temporal patterns across outcome measures. MSES and 6MWD demonstrated significant group × time interactions (both *P* < 0.001), while IPAQ and CQQC showed consistent group differences without significant interactions (*P* = 0.228 and *P* = 0.158, respectively). This pattern provides important insights into how Guozhuang dance exerts its therapeutic effects.

The significant interaction effects for MSES and 6MWD indicate that benefits on exercise self-efficacy and functional capacity accumulate progressively over time. This aligns with Bandura's self-efficacy theory (23), where mastery experiences develop through repeated successful performance. As patients continued practicing Guozhuang dance, accumulated mastery experiences led to progressively greater improvements in self-efficacy. Similarly, cumulative physiological adaptations from regular aerobic exercise—including enhanced cardiovascular function and improved endurance—explain the time-dependent improvements in 6MWD (31, 32). These findings emphasize that sustained participation is essential, as early discontinuation would forfeit cumulative gains.

In contrast, the absence of interaction effects for IPAQ and CQQC indicates that Guozhuang dance established immediate and sustained benefits in physical activity and quality of life from the outset. The engaging nature of Guozhuang dance—incorporating music, social interaction, and culturally relevant movements—may provide immediate psychological benefits that translate into enhanced quality of life early in the program (12, 35, 40). The structured group format may also establish consistent behavioral patterns early, leading to stable increases in physical activity throughout the intervention period.

From a clinical perspective, these differential patterns suggest that cardiac rehabilitation programs should be designed with both short-term and long-term goals. While patients may experience immediate benefits in quality of life and activity levels, achieving substantial gains in self-efficacy and functional capacity requires sustained engagement. Healthcare providers should emphasize long-term adherence and set realistic expectations regarding the timeframe for different improvements.

## 5 Conclusion

This study applied Guozhuang Dance as an intervention for patients with coronary heart disease following PCI, and found that it effectively improved patients’ post-procedural exercise self-efficacy, quality of life, and physical activity levels. The intensity of Guozhuang Dance is well-suited for cardiac rehabilitation in post-PCI patients. Additionally, its advantages—including ease of implementation, high entertainment value, and strong safety profile—make it a promising option for cardiac rehabilitation.

However, this study had several limitations. First, while the “Xiao Daka” WeChat mini-program facilitated self-monitoring, it relied on self-reported data rather than objective measures such as accelerometers or heart rate monitors, which are increasingly standard in contemporary exercise trials. Second, due to the behavioral nature of the intervention, blinding of participants and intervention staff was not feasible; although we assigned participants from separate wards to minimize contamination, this lack of blinding is an inherent limitation of behavioral interventions and may have influenced outcomes. Third, potential compliance bias cannot be fully excluded, as completers may have differed from non-completers in unmeasured factors (e.g., motivation, social support) that could independently affect outcomes, despite consistency between ITT and per-protocol results. Fourth, the intervention duration was limited to 12 weeks, potentially limiting assessment of long-term sustainability. Finally, the relatively small sample size may affect generalizability. Future studies should incorporate remote monitoring technologies, extend the intervention period, and increase sample size to further validate the safety and long-term effectiveness of Guozhuang Dance in cardiac rehabilitation.

## Data Availability

The original contributions presented in the study are included in the article/Supplementary Material, further inquiries can be directed to the corresponding author.

## Supplementary material

Supplementary Data Sheet 1 Guozhuang dance exercise program.

Supplementary Data Sheet 2 Pilot study results.

Supplementary Data Sheet 3 Health handbook.

Supplementary Data Sheet 4 Per-protocol analysis.
