# Supplementary material for: The effects of Guozhuang dance on exercise self-efficacy in coronary heart disease patients following percutaneous coronary intervention: a randomized controlled trial
Source: Front Cardiovasc Med. 2026 Jan 19;12:1688894. doi: 10.3389/fcvm.2025.1688894 (PMC12862062; doi:10.3389/fcvm.2025.1688894)

# Control Group ：

Routine post-PCI care: Upon discharge, patients received a handbook titled "Secondary Prevention Knowledge After Percutaneous Coronary Intervention for Coronary Heart Disease," which provided education on post-procedural precautions, including medication use and adverse reactions, diet, wound care, and follow-up appointment schedules. Patients were advised to engage in aerobic exercise: at least 150 minutes of moderate-intensity aerobic exercise per week was recommended. Exercise options included walking, Tai Chi, Baduanjin (Eight-Section Brocade), calisthenics, jogging, and swimming. Patients' exercise activities were recorded, with examples such as: "I walk for 30 minutes per day, 5 times per week"; "I practice Tai Chi for 50 minutes per day, 3 times per week"; "I swim for 40 minutes per day, 4 times per week."

# Intervention Group——Guozhuang Dance Exercise Program

| Module | Exercise Objectives | Exercise Content | Exercise Characteristics |
| --- | --- | --- | --- |
| Warm-up (≈5–10 min) | To activate large muscle groups, promote coordination and joint flexibility, increase body temperature, and prevent joint injuries. | 1) Basic posture: stand naturally, gently raise shoulders and chest, tilt head left and right. 2) Shoulder rotation: slow shoulder rotation and neck extension. 3) Spinal stretch and chest expansion. 4) March in place and side steps. 5) Arm swings and leg extensions. | Movements involve the neck, shoulders, waist, knees, and ankles. Promote systemic circulation, flexibility, and muscle activation. |
| Guozhuang Dance (≈30–40 min) | To enhance cardiopulmonary function, improve physical fitness, and increase exercise satisfaction and self-efficacy. | Group 1: Hand claps, footwork, body movements in a rhythmic sequence. Group 2: Step-touch with arm swings, claps over head and chest. Group 3: Hip sways, coordinated steps with arm raises. Movements are selected from the first 4 combinations of the dance “Zha xi Leyang Le”. | Open-limb movements with moderate intensity (HR 120–150 bpm), promote aerobic endurance and coordination. Music-guided rhythm enhances motivation. |
| Stretching (≈5–10 min) | To relieve muscle tension, prevent soreness and fatigue, and reduce joint injury risk after exercise. | 1) Deep breathing and full-body stretch. 2) Shoulder and back loosening. 3) Chest and abdominal extension. 4) Hamstring and quadriceps stretch. 5) Calf and Achilles tendon stretch. | Focused on relaxation and recovery, targeting shoulders, chest, abdomen, and lower limbs. Promotes full-body release. |


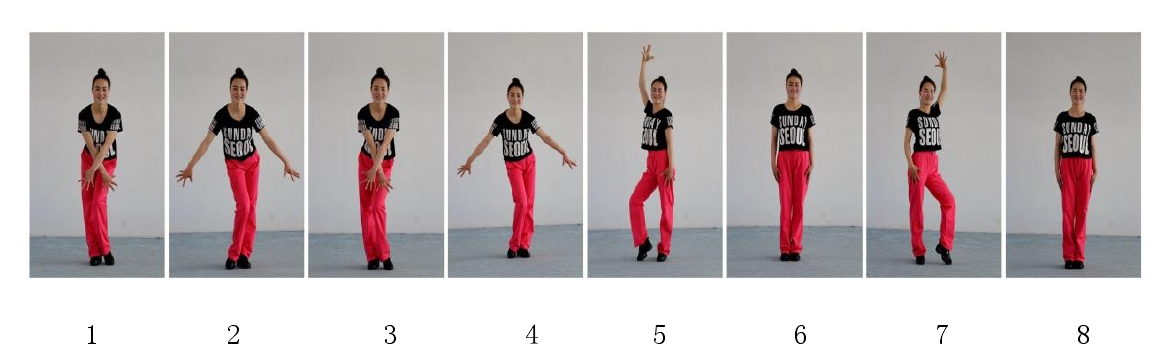


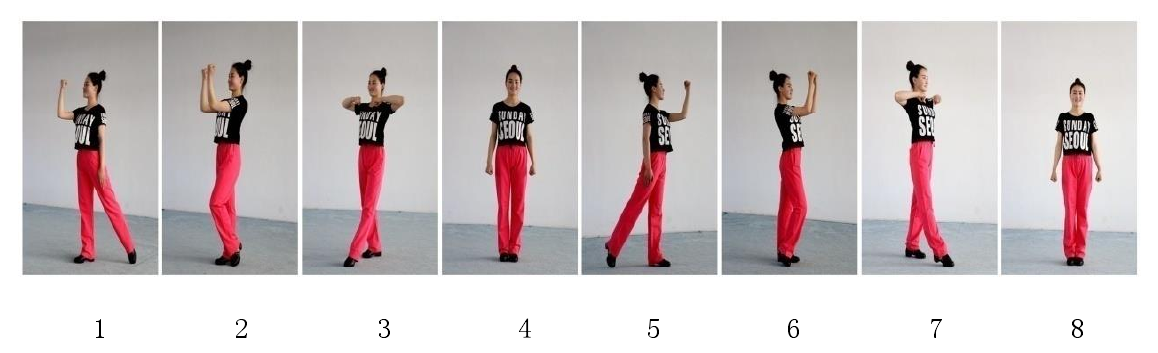


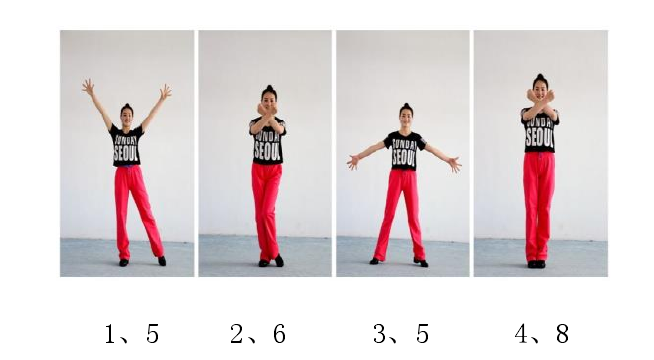


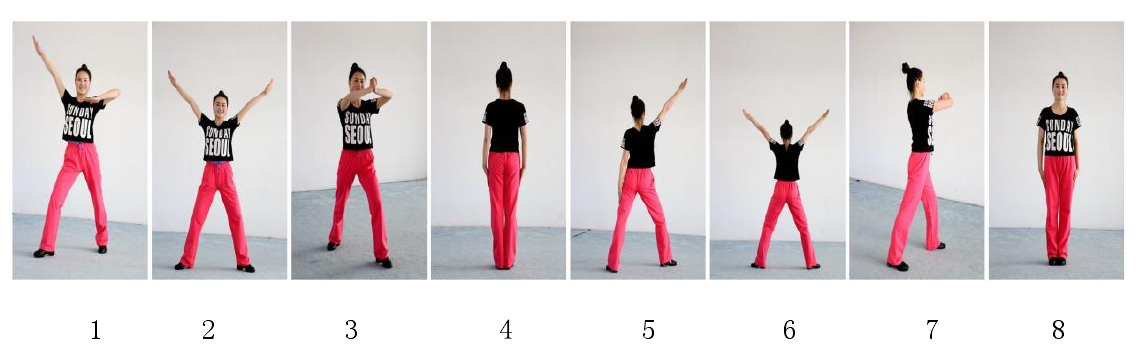

Supplement: Supplementary Data Sheet 1 — Guozhuang dance exercise program. [file Datasheet1.docx]
