# Supplementary material for: The effects of Guozhuang dance on exercise self-efficacy in coronary heart disease patients following percutaneous coronary intervention: a randomized controlled trial
Source: Front Cardiovasc Med. 2026 Jan 19;12:1688894. doi: 10.3389/fcvm.2025.1688894 (PMC12862062; doi:10.3389/fcvm.2025.1688894)
Supplement: Supplementary Data Sheet 2 — Pilot study results. [file Datasheet2.docx]

Pilot Trial

To enhance the scientific and practical nature of this study and to obtain feedback from patients for timely optimization of the intervention program, a preliminary trial was conducted on 12 PCI patients who met the exclusion criteria and had signed informed consent forms.

(1) Study Subjects for the Preliminary Trial

**Inclusion Criteria**:(a)Patients aged 18-75 years who are undergoing their first PCI procedure, with 1-2 stents implanted during surgery.(b)Patients with heart function classified as New York Heart Association (NYHA) class I or II.(c)Stable condition without communication difficulties.(d)Patients who have not previously participated in Guozhuang dancing and are willing to participate in the study.(e)Patients who meet the 2020 Guidelines for Stable Coronary Artery Disease Diagnosis and Treatment in Primary Healthcare in China.

**Exclusion Criteria**(a)Patients with mental, personality, or intellectual disabilities.

(b)Patients with atrioventricular block, atrial fibrillation, severe heart failure, or malignant tumors. (c)Patients with chronic obstructive pulmonary disease (COPD), asthma, or other pulmonary diseases that may interfere with regular physical activity.

(d)Patients who have difficulty with mobility or cannot attend follow-up appointments.

(2) Specific Implementation of the Preliminary Trial

The 12 patients were randomly assigned into two groups: a control group (n=6) and an experimental group (n=6).

Control Group: Standard post-PCI care, routine physical rehabilitation, and follow-up care after discharge.

Intervention Group: In addition to the standard post-PCI care, the patients participated in a 4-week Guozhuang dance intervention.Throughout the pilot trial, the research team continuously tracked and recorded the patients' Exercise Self-Efficacy (ESE) to inform future adjustments to the study protocol. At the end of the 4-week intervention, both groups were followed up in the outpatient clinic to collect relevant data.

(3) Evaluation Metrics for the Preliminary Trial

The Exercise Self-Efficacy (ESE) scores of the participants were recorded on the day of discharge and 4 weeks after the intervention.

(4) ESE Scores of the Two Groups in the Preliminary Trial

The ESE scores of the two groups at the beginning and end of the trial are presented in Table 1. A statistically significant difference in the scores between the two groups was observed (P < 0.05), as shown in Table 2.

Table 1 General Demographic Data of Study Participants in the Pilot Trial

| Group | ID | Gender | Age | Education Level | On the Day of Discharge | 4 Weeks Post-Intervention | | Difference |
| --- | --- | --- | --- | --- | --- | --- | --- | --- |
| Control Group | A | Male | 67 | Associate Degree | 21 | 25 | 4 | |
|  | B | Male | 53 | High School | 43 | 50 | 7 | |
|  | C | Female | 63 | Elementary School   | 31 | 37 | 6 | |
|  | D | Female | 66 | High School | 34 | 41 | 7 | |
|  | E | Female | 66 | Elementary School | 65 | 70 | 5 | |
|  | F | Male | 46 | Associate Degree | 39 | 46 | 7 | |
| Intervention Group | G | Female | 56 | Junior High School | 26 | 44 | 18 | |
|  | H | Male | 64 | High School | 31 | 43 | 12 | |
|  | I | Female | 61 | High School | 35 | 48 | 13 | |
|  | J | Male | 61 | High School | 37 | 50 | 13 | |
|  | K | Male | 56 | Junior High School | 39 | 48 | 9 | |
|  | L | Female | 69 | Junior High School | 26 | 45 | 19 | |

Table2 Comparison of Exercise Self-Efficacy Score Differences Between the Two Groups in the Pilot Trial

|  | Pre-intervention | Post-intervention |
| --- | --- | --- |
| Control Group | 33.83±13.70 | 39.83±13.89 |
| Intervention Group | 32.33±5.57 | 46.33±2.73 |
| *t* | -4.899 | |
| *P* | 0.003 | |

**(5) Patient Feedback and Expert Recommendations**

Based on feedback from the patients involved in the preliminary trial and expert suggestions, it was indicated that the proposed intervention protocol is reasonable, with strong clinical applicability. Patients expressed a high level of acceptance toward the Guozhuang dance, and several recommendations were made, which have been incorporated into the Guozhuang dance intervention protocol. These suggestions are summarized in Table 3.

Table 3: Recommendations for Revision of the Pilot Trial Protocol

|  | Before Modification | After Modification | Reason for Modification |
| --- | --- | --- | --- |
| Patient | No background music for Guozhuang dance | Provide patients with a dance music library, including "吹起羌笛跳锅庄","天路", "草原上的格桑花", etc. | Patient feedback indicated a lack of rhythm in the Guozhuang dance. |
| Cardiology Expert | Exclusion criteria: Exclude patients with severe heart failure, malignant tumors, asthma, or other conditions that may affect walking or breathing exercises. | Add malignant hypertension to the exclusion criteria. | Malignant hypertension has exercise contraindications. |
