# Supplementary material for: The effects of Guozhuang dance on exercise self-efficacy in coronary heart disease patients following percutaneous coronary intervention: a randomized controlled trial
Source: Front Cardiovasc Med. 2026 Jan 19;12:1688894. doi: 10.3389/fcvm.2025.1688894 (PMC12862062; doi:10.3389/fcvm.2025.1688894)
Supplement: Supplementary Data Sheet 3 — Health handbook. [file Datasheet3.doc]

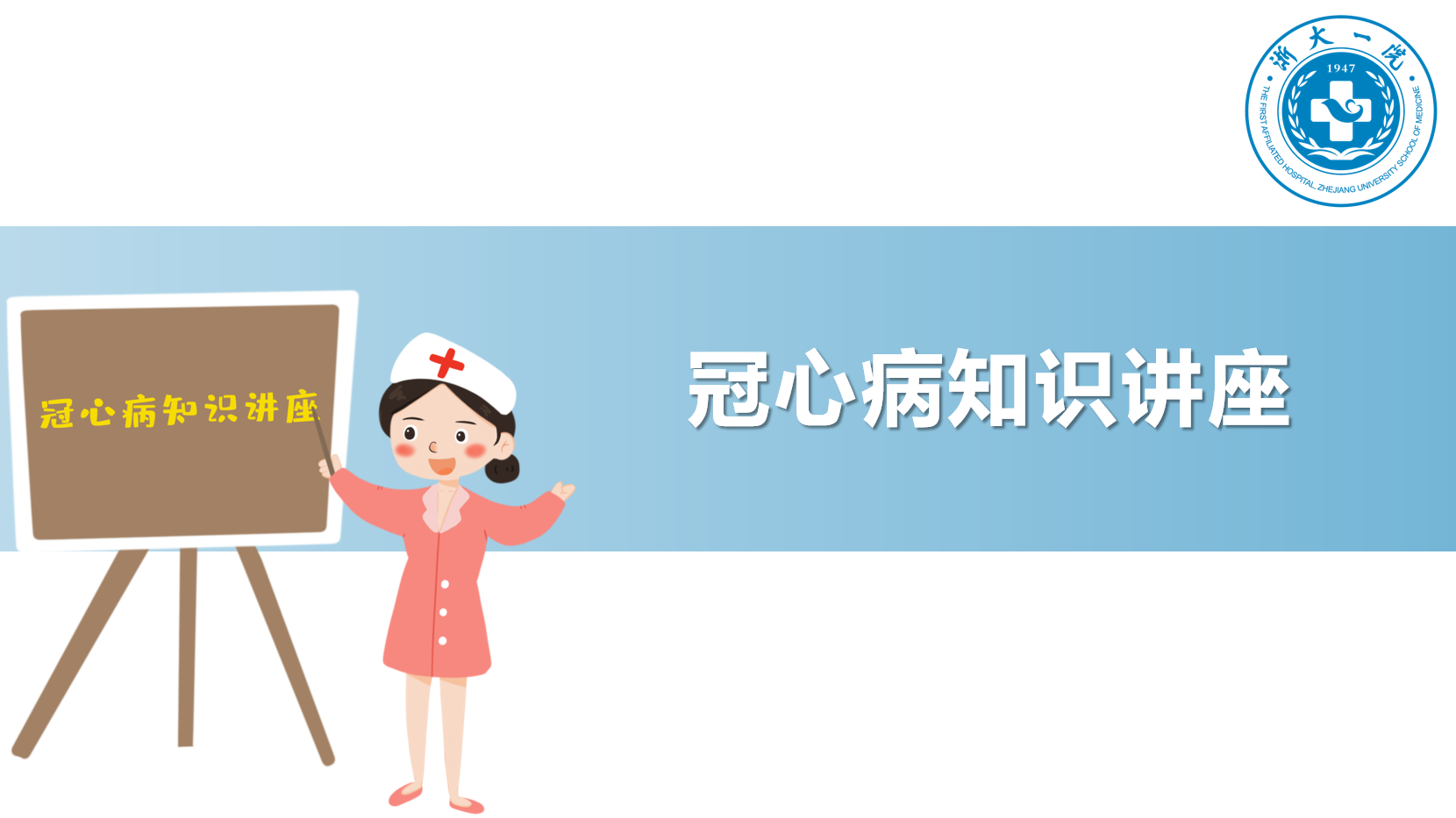


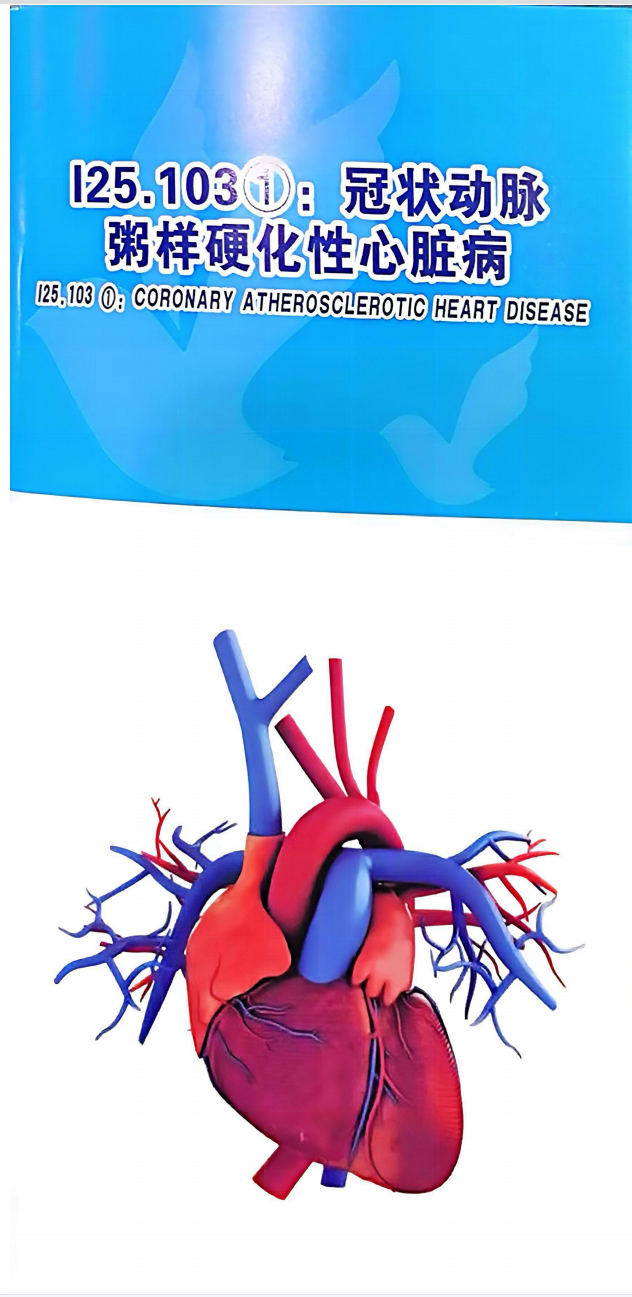

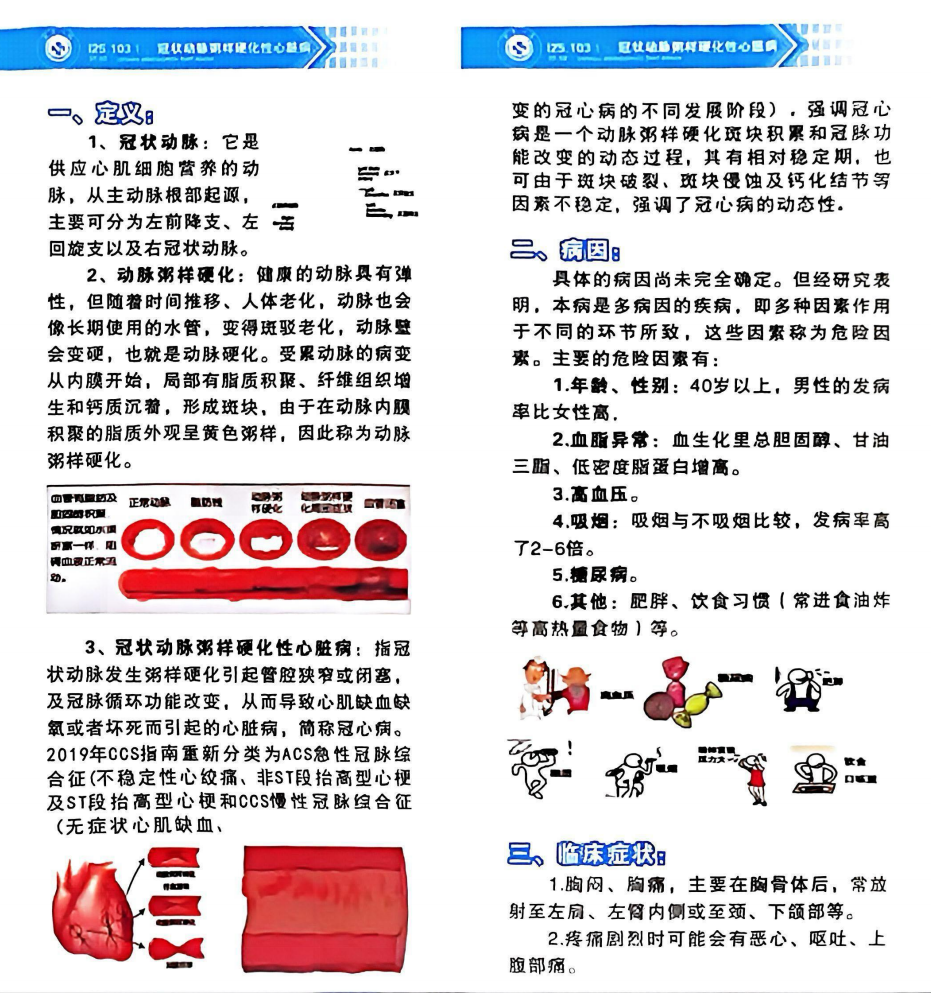

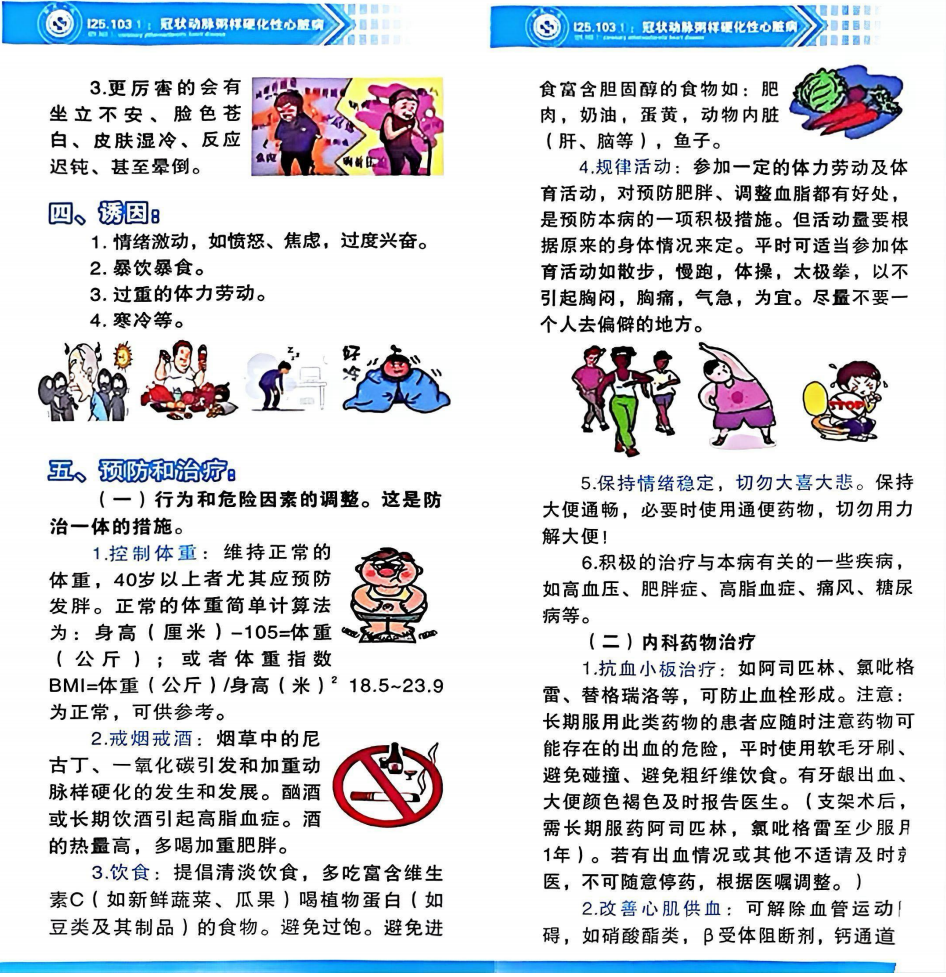


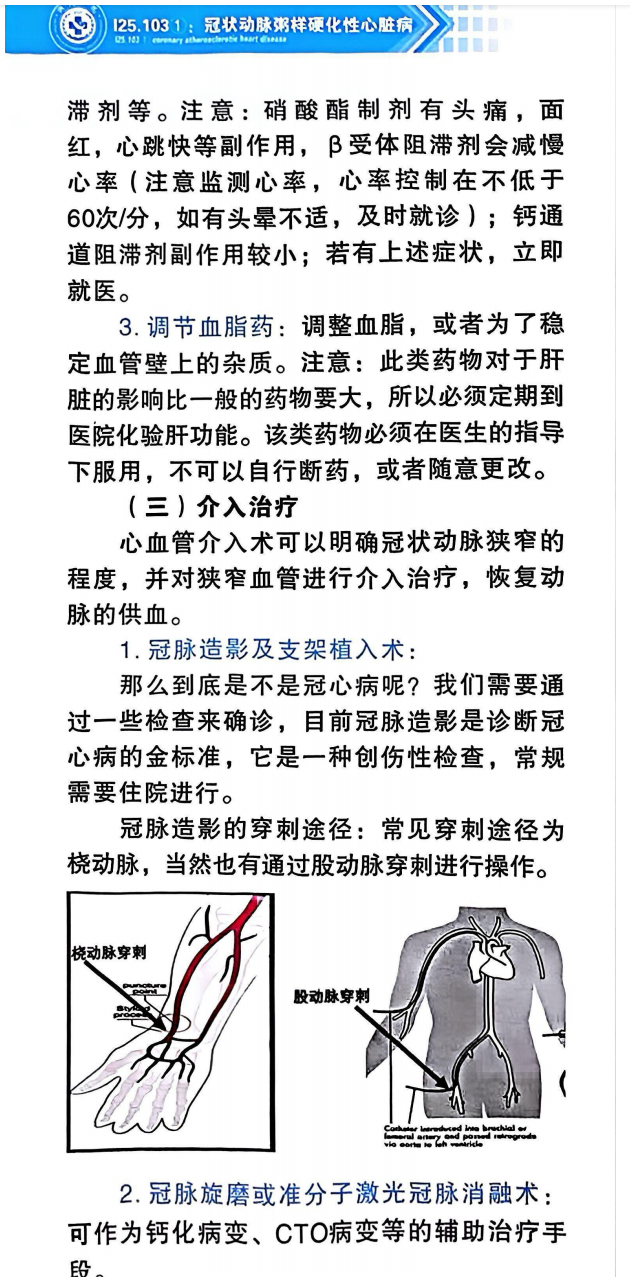

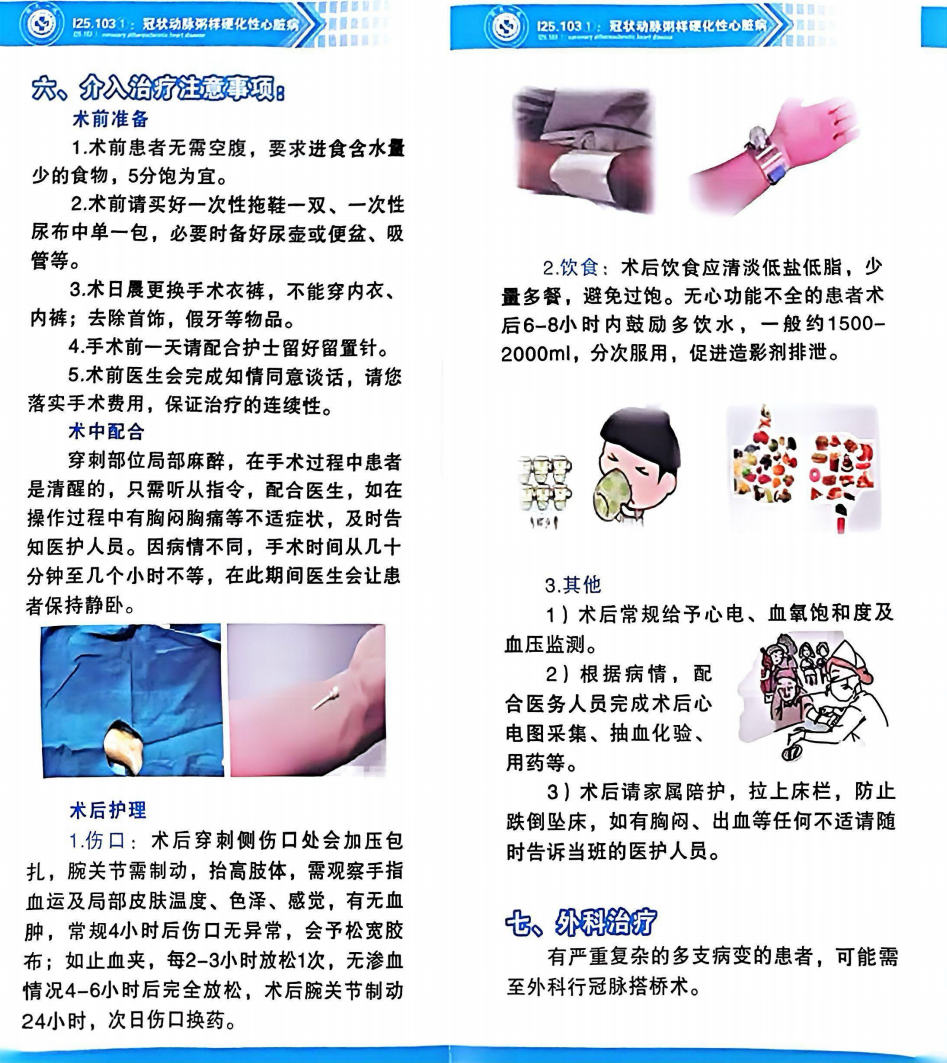

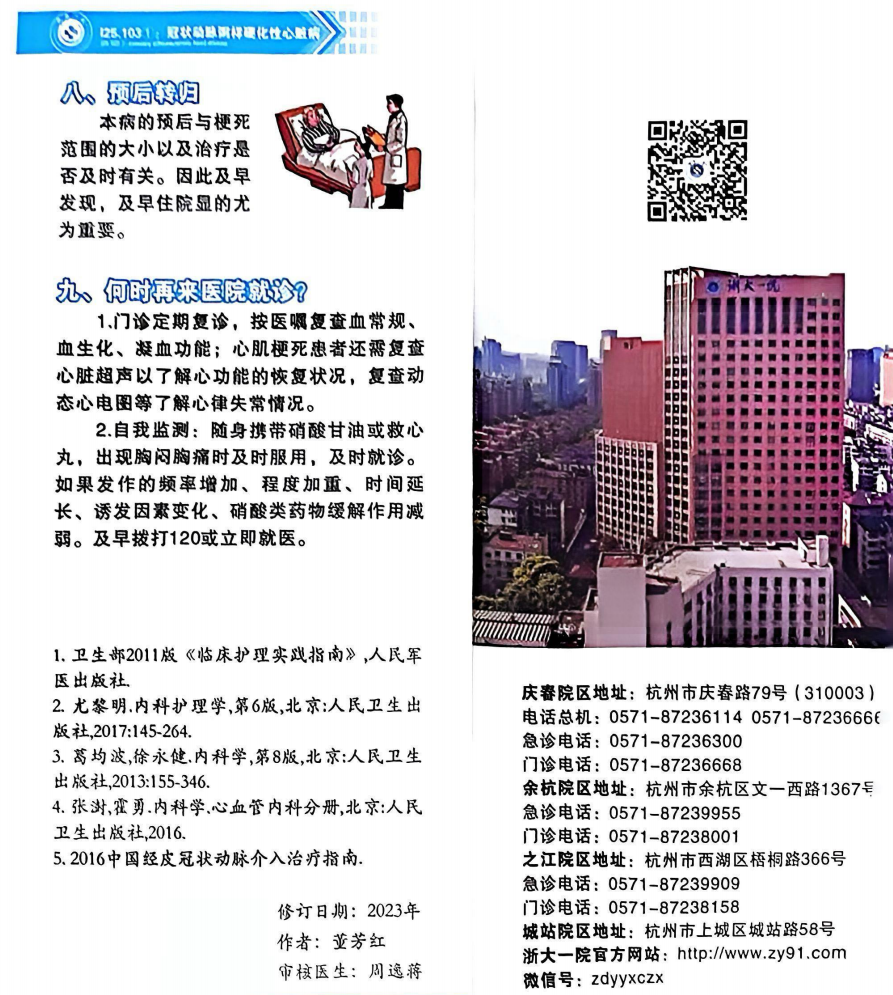


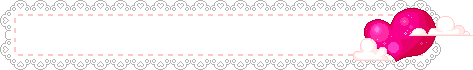
**冠心病健康教育手册**

一、什么是冠心病？

二、冠心病临床症状有哪些？

三、心肌梗塞是怎样形成的

四、哪些人易患冠心病

五、肥胖与冠心病有何关系？

六、糖尿病与冠心病有何关系？

七、气候变化与冠心病有何关系？

八、为什么Ａ型性格的人易患冠心病？

九、诊断冠心病的方法有哪些？

十、冠心病病人都会有心电图的改变吗?有心电图改变就一定是冠心病吗？

十一、有典型心绞痛发作，但发作时心电图无缺血性改变，能否诊断冠心病？

十二、为什么有的病人在劳累时发生心绞痛，而有的则在休息或睡眠时发生？

十三、怎样早期发现冠心病？

十四、为及早发现冠心病，40岁以上的人应定期做哪些检查？

十五、冠心病病人在冬春季节中要注意什么？

十六、冠心病病人怎样选择食物？

十七、冠心病病人为何应少吃食盐？

十八、冠心病病人应如何对待吸烟和喝酒、咖啡及茶？

十九、老年冠心病病人能否拔牙？

二十、有心肌梗死的病人为何不要饱餐和饱餐后沐浴？

二十一、冠心病病人在运动中要注意什么？

二十二、老年冠心病患者如何康复锻炼？

二十三、冠心病病人外出旅游要注意些什么？

二十四、控制冠心病的关键是什么？

二十五、预防冠心病的措施

二十六、治疗冠心病的常用药物有哪几类？

二十七、冠心病急性发作时的治疗

二十八、冠心病发作时怎样使用保健盒进行自救？

二十九、冠心病的药物 介入 手术搭桥治疗

三十、中医对冠心病的治疗手段或处方用药

三十一、冠心病心肝失调型药膳

**一、什么是冠心病？**

冠心病是冠状[动脉粥样硬化性心脏病的简称。是指供给心脏营养物质的血管——冠状动脉发生严重粥样硬化或痉挛，使冠状动脉狭窄或阻塞，以及血栓形成造成管腔闭塞，导致心肌缺血缺氧或梗塞的一种心脏病，亦称缺血性心脏病。](http://www.cnkang.com/jbdq/nk/xnk/zyh/Index.html)[冠心病是动脉粥样硬化导致器官病变的最常见类型，也是危害中老年人健康的常见病。本病的发生与冠状动脉粥样硬化狭窄的程度和支数有密切关系，但少数年轻患者冠状](http://www.cnkang.com/jbdq/nk/xnk/gxb/Index.html)[动脉粥样硬化虽不严重，甚至没有发生粥样硬化，也可以发病。也有一些](http://www.cnkang.com/jbdq/nk/xnk/zyh/Index.html)[老年人冠状](http://www.cnkang.com/dzjk/lnjk/Index.html)[动脉粥样硬化性狭窄虽较严重，并不一定都有胸痛、心悸等冠心病临床表现。因此，](http://www.cnkang.com/jbdq/nk/xnk/zyh/Index.html)[冠心病的发病机理十分复杂，总的来看，以器质性多见，冠状动脉痉挛也多发生于有粥样硬化的冠状动脉。](http://www.cnkang.com/jbdq/nk/xnk/gxb/Index.html)

**二、冠心病临床症状有哪些？**

1．心绞痛型：表现为胸骨后压榨感,闷胀感,持续3到5分钟,常发散到左侧臂部, 休息和含化硝酸甘油缓解。

2．心肌梗塞型：疼痛部位与以前心绞痛部位一致,但持续更久,疼痛更重,休息和含化硝酸甘油不能缓解。

3．无症状性心肌缺血型：有广泛的冠状动脉阻塞,心肌缺血却没有心绞痛发作。

4．心力衰竭和心律失常型：部分患者出现心力衰竭的表现,如气紧,水肿,乏力等,还有各种心律失常。

5．猝死型：指由于冠心病引起的不可预测的突然死亡,在急性症状出现以后1小时内发生心脏骤停所致。

**三、心肌梗塞是怎样形成的**

心肌梗塞的主要病因是冠状动脉粥样硬化，偶为冠状动脉栓塞 、炎症、先天性畸形等。当冠状动脉内膜下斑块造成管腔狭窄，血流通过缓慢，心肌供血不足，而侧支循环尚未充分建立时，一旦血供进一步急剧中断，使心肌严重而持久地急性缺血达1小时以上，即可发生心肌梗塞。

此外，在冠状动脉粥样硬化基础上，冠脉内膜增厚，斑块合并出血、溃疡、钙化等使血管内膜粗糙不平，血小板易于聚集、吸附，从而使管腔内血栓形成或诱发冠脉血管持续痉挛，最终导致冠状动脉完全闭塞而发生心肌梗塞。再如休克、脱水、大出血、外科手术或严重心律失常等可致心排出量骤降，冠状动脉灌流量锐减，均可使心肌供血急剧减少或中断。其它如重体力活动、情绪过分激动或血压剧升，心室负荷明显加重；儿茶酚胺分泌增多，心肌需氧量猛增，冠状动脉供血相对不足，也都是心肌梗塞形成的重要因素

**四、哪些人易患冠心病**

1．冠心病多见于40岁以上的中老年人，49岁以后进展较快，心肌梗塞与冠心病的猝死发病与年龄成正比。年轻的男性患者比年轻的女性患者多，但绝经后的女性以及年过六十的妇女，其危险就几乎与男性相等了，甚至大于男性。老年人心脏病发作的可能性较高。

2．吸烟者罹患这种疾病的可能性比不吸烟者至少大2倍。且与每日吸烟支数成正比。在三十五到四十五岁的年龄层中，吸烟者死于冠状动脉病的人数，是不吸烟者死于冠状动脉病人数的5倍以上。香烟可损坏动脉，加速动脉粥样硬化的形成并使心脏缺氧。

3．饮食：常进较高热量的饮食、较多的动物脂肪、胆固醇者易患本病。同时食量多的人也易患本病。

4．如果患有高血压或糖尿病，将增加患冠状动脉病的危险。男性糖尿病患者罹患冠状动脉病的机率，是其他男性的2倍，女性糖尿病患者罹患冠状动脉病的机率，则是其他妇女的5倍。有高血压表示心脏需加倍工作。心脏病发作的机会也越高。血压升高是冠心病的独立危险因素。高血压病患者患本病者是血压正常者的４倍。

5．冠心病具有家族遗传性，若家族中有人患上冠心病，就更容易有心脏病发作。

6．体重超重，患冠心病的可能性就比体重正常的人要大。体重超重多过20%的人心脏病发作的可能性比体重健康的人高三倍。

7．如果是从事经常坐着不动的工作，罹患冠状动脉病的可能性就比从事包括体力劳动在内的工作者要大。缺少运动心脏不强壮者得心脏病发作的机会比健康者高出两倍。

8．如果是女性，已年过35岁，并且在服用避孕药，就较采用其他方式避孕的妇女容易罹患冠心病。

9．高血脂：由于遗传因素，或脂肪摄入过多，或脂质代谢紊乱而致血脂异常。如总胆固醇、甘油三酯、低密度脂蛋白胆固醇增高与本病有关，而高密度脂蛋白的降低易患本病。血胆固醇量每100毫升最好低过200毫克（5.2mmol/L）。含量越高，危险性就越高。

10．持久的精神压力是公认的致病因素之一。脑力劳动者大于体力劳动者，经常有紧迫感的工作较易患病。

以上因素中，血压过高、体重超标、胆固醇过高是导致冠心病的最大危险因素。

**五、肥胖与冠心病有何关系？**

许多资料表明，冠心病患者的平均体重较非冠心病患者为高，肥胖者冠心病的发病率较高， 尤其是短期内发胖或重度肥胖者发病率更高。这是因为：

1．肥胖者摄取过多的热量，在体重量增加的同时，使心脏负荷和血压均升高，从而增加心肌耗氧量；

2．高热量的饮食习惯，使胆固醇、甘油三酯和血压升高，促使冠状动脉粥样硬化的形成和加重；

3．肥胖者体力活动减少，妨碍了冠状动脉粥样硬化侧支循环的形成；

4．肥胖者常使胰岛素的生物学作用被削弱，即这些人的机体对胰岛素产生抵抗，容易形成糖尿病。

糖尿病、高脂血症、高纤维蛋白原血症无一不是动脉粥样硬化的危险因素，于是冠心病便接踵而至。

**六、糖尿病与冠心病有何关系？**

糖尿病是一种全身性代谢紊乱性疾病，容易引起冠心病。研究表明，糖尿病患者，无论男女，不同年龄组，其心血管病的发病率都是糖尿病组高于非糖尿病组。对两性来说，男性具有较强的发病危险，但经年龄调整和控制冠心病其它危险因素后，女性糖尿病患有冠心病的发病危险明显高于男性，甚至有报道，糖尿病是女性冠心病的独立危险因素。

糖尿病患者冠心病发病率增高的原因尚不十分清楚，但糖尿病容易引起动脉粥样硬化已被公认。多数学者认为，肥胖、高血压、高脂蛋白血症、高血糖、高纤维蛋白血症（即胰岛素抵抗综合征），这些内容密不可分。肥胖使机体对胰岛素产生抵抗，为了保证血糖的水平正常，胰岛β细胞必须分泌较正常人高几倍、甚至几十倍的胰岛素，形成高胰岛素血症，但最终又导致了血糖升高、血甘油三酯水平升高、ＨＤＬ－Ｃ降低、血浆纤维蛋白原升高，这些都是动脉粥样硬化的危险因素。

胰岛素本身也有促进动脉粥样硬化的作用，因此，口服磺尿类降糖药或注射胰岛素等通过不同途径使血中胰岛素水平提高，这就有可能进一步加重硬化血管的病变。此外，糖尿病患者并发冠心病时， 冠心病的某些临床症状出现的较迟或被掩盖。因为糖尿病性神经病变可累及神经系统的任何一部分，特别是神经末梢，当病人的神经末梢受损时，痛阈升高，即使发生了严重的心肌缺血，疼痛也较轻徽而不典型，甚至没有心绞痛症状，无痛性心肌梗塞的发生率高，而且休克、心力衰竭、猝死的并发症也较多，预后较严重。

**七、气候变化与冠心病有何关系？**

气候寒冷的天气或冬春季节， 冠心病心绞痛和心肌梗塞的发病率就会增加。三个与冠心病有关的最优因子为：气温、日变差（相邻两日的日平均气温之差）和平均风速。持续低温、阴雨和大风天气容易发病。此外，在年平均气压高低不同时期亦有显著差别，以气压低时发病高。

在寒冷、潮湿和大风天气，冠心病发病率高是因为寒冷刺激，特别是迎风疾走，易使交感神经兴奋，使心率加快，血压升高，体循环血管收缩，外周阻力增加，心肌耗氧量增多，同时，也可诱发冠状动脉痉挛，使管腔持续闭塞，或挤压斑块使内膜损伤，血小板聚集，血栓形成使管腔急性堵塞，也可导致急性心肌梗塞。

因此，在高发季节里，冠心病患者应注意御寒保暖，减少户外活动，以防疾病发生。

**八、为什么Ａ型性格的人易患冠心病？**

Ａ型性格者冠心病发病率是Ｂ型性格的２倍，Ａ型性格占冠心病人数的70.9％。 那么，什么是Ａ型性格，Ａ型性格的人为什么易患冠心病呢？美国心脏病专家弗里德曼和罗林曼把人的性格分为Ａ、Ｂ两种类型。

 具有Ａ型性格的人动作匆忙，办事的节奏快，有时间紧近感，争强好胜，遇到困难也不罢休，对任何事情都有一种不满足感。一件事情没有做完，又去做另一件事情，四处奔忙。这种人雄心勃勃，脾气暴躁，干练利索，性格外向。他们常常为一些小 事就可以大发雷霆，虽然有事业心，但对周围的人怀有“敌意”。另外，由于Ａ型性格的人过于追求事业和功名，却常常忽视个人的健康，他们不会享受生活的乐趣，不懂得如何照顾自己，常使自己整天处在紧张和压力之中。恰恰相反，具有Ｂ型性格的人，他们慢条斯理，不慌不忙，随和易处，没有争强好胜的压力，紧张之后能愉快地休息。

近年来研究表明，冠心病与心理紧张有关。弗里德曼与罗林曼通过大量研究人的心理活动与疾病的关系后发现，心脏病人几乎都是些思想敏锐而雄心勃勃的人，这正是Ａ型性格的人。如果人长期地、反复地处于紧张状态中，在这些因素作用下，极易形成冠心病。

**九、诊断冠心病的方法有哪些？**

1．临床表现：心绞痛是冠心病的主要临床症状，根据心绞痛发作时的部位、性质、诱因、持续时间、缓解方式等特点和伴随症状及体征便可鉴别心绞痛和心肌梗塞，可以说，典型的症状和体征对冠心病心绞痛和心肌梗塞的诊断至关重要。

2．心电图：心电图是冠心病诊断中最早、最常用和最基本的诊断方法，心电图使用方便，易于普及，当患者病情变化时便可及时捕捉其变化情况，并能连续动态观察和进行各种负荷试验，以提高其诊断敏感性。无论是心绞痛或心肌梗塞，都有其典型的心电图变化。

3．核素心肌显像：根据病史，心电图检查不能排除心绞痛时可做此项检查。核素心肌显像可以显示缺血区、明确缺血的部位和范围大小。结合运动试验再显像，可提高检出率。

4．冠状动脉造影：是目前冠心病诊断的“金标准”。可以明确冠状动脉有无狭窄、狭窄的部位、程度、范围等，并可据此指导进一步治疗所应采取的措施。结合左心室造影，可以对心功能进行评价。

5．超声和血管内超声：心脏超声可以对心脏形态、室壁运动以及左心室功能进行检查，是目前最常用的检查手段之一。血管内超声可以明确冠状动脉内的管壁形态及狭窄程度，是一项很有前景的新技术。尤其适用于造影剂过敏，不能做冠状动脉造影者。

6．心肌酶学检查：是急性心肌梗塞的诊断和鉴别诊断的重要手段之一。临床上根据血清酶浓度的序列变化和特异性同工酶的升高等肯定性酶学改变可明确诊断为急性心肌梗塞。

7．心血池显像：可用于观察心室壁收缩和舒张的动态影像，对于确定室壁运动及心功能有重要参考价值。

**十、冠心病病人都会有心电图的改变吗?有心电图改变就一定是冠心病吗？**

当你心前区经常感到闷痛而心电图结果又提示正常时，你可能会放心地认为自己没有冠心病,其实切不可掉以轻心。因为一张正常的心电图，并不能排除心脏病的存在。

要知道心电图检查对冠心病的诊断并不是一个非常敏感的方法。冠心病在非发病时期，其心电图检出率仅是30%～50%，而50%以上的病人心电图表现正常。

另外，心脏及冠状动脉循环有较大的代偿能力，在休息和平静时有时不易检出异常，往往需要通过增加心脏负荷的运动试验，才能发现心电图的真正改变。

但当你看到心电图报告上有某些医学术语时，也不要忧心忡忡，以为自己得了心脏病，因为单凭一份心电图不能对病轻易下冠心病的诊断，因为有许多疾病如心肌病、心肌炎、自主神经功能紊乱等，都可以产生与冠心病相同的心电图表现。

所以心电图对冠心病的诊断不是一个非常特异的方法。尽管心电图检查对冠心病的诊断是一项重要的临床参考依据，但并非唯一的诊断标准，因此，临床上对冠心病的诊断必须根据病史、症状和某些特殊检查，进行全面综合判断才可能做出。

**十一、有典型心绞痛发作，但发作时心电图无缺血性改变，能否诊断冠心病？**

冠心病心绞痛主要是一种症状诊断。临床上，病人只要有典型的心绞痛发作，无论心电图是否有相应的缺血改变，即可诊断为冠心病。

有典型劳力性心绞痛的病人，如发作时心电图仍正常，为估价冠状动脉储备能力，可做蹬车或踏板运动试验，或201铊运动试验。另外，运动试验还有助于对病人冠脉病变程度作出评价。

**十二、为什么有的病人在劳累时发生心绞痛，而有的则在休息或睡眠时发生？**

心绞痛是一种由于心肌暂时缺血、缺氧引起的，以发作性胸痛或胸部不适为主要表现的临床综合征。通常有冠状动脉供血绝对减少和心肌需氧量突然增加两种情况。

心绞痛最常见的基本病因是冠状动脉粥样硬化引起的冠状动脉大分支管腔狭窄。一般情况下，安静时狭窄的冠状动脉可以向心肌提供其所需的血氧，但当进行较大负荷的劳动或情绪激动时，心肌耗氧量骤然增加，而发生粥样硬化的冠状血管弹性减退，不能充分扩张以向心肌供应足够的血液，造成心肌缺氧而引起心绞痛。

在临床上我们还常碰到另外一类病人，他们常于休息时，心肌需氧量并未增加的情况下发生心绞痛，在劳动时反而很少发生或不发生。这是因为此类心绞痛的病人于休息时，冠状动脉的大分支常有自发的或诱发的痉挛。冠状动脉造影清楚地证明了这一点。休息时发生心绞痛者，并不意味着其冠状动脉粥样硬化的程度一定比劳力性心绞痛者严重。冠状动脉痉挛也可发生于造影完全正常的冠状动脉，但一般都有不同程度的粥样硬化基础。冠状动脉痉挛的发生，可能与自主神经功能紊乱和动脉粥样斑块部位的动脉壁对神经体液因素的影响过度敏感有关。前列腺素的一些中间代谢产物也可能引起冠状动脉痉挛。

**十三、怎样早期发现冠心病？**

冠心病是中老年人的常见病和多发病，处于这个年龄阶段的人，在日常生活中，如果出现下列情况，要及时就医，尽早发现冠心病。

1．劳累或精神紧张时出现胸骨后或心前区闷痛，或紧缩样疼痛，并向左肩、左上臂放射，持续３-５分钟，休息后自行缓解者。

2．体力活动时出现胸闷、心悸、气短，休息时自行缓解者。

3．出现与运动有关的头痛、牙痛、腿痛等。

4．饱餐、寒冷或看惊险影片时出现胸痛、心悸者。

5．夜晚睡眠枕头低时，感到胸闷憋气，需要高枕卧位方感舒适者；熟睡、或白天平卧时突然胸痛、心悸、呼吸困难，需立即坐起或站立方能缓解者。

6．性生活或用力排便时出现心慌、胸闷、气急或胸痛不适。

7．听到噪声便引起心慌、胸闷者。

8．反复出现脉搏不齐，不明原因心跳过速或过缓者。

**十四、为及早发现冠心病，40岁以上的人应定期做哪些检查：**

如果检验结果不正常或有其他的易患冠心病的危险因素，应该每五年作一次或更多次血胆固醇检查。

每年作一次血压检查。

每年作一次血糖检查。

若属于冠心病的高危人群，就要请医生查看是否需要接受心电图检查。若需要进一步的检查，医生会安排做一项运动试验以测出在踩固定脚车或踩运动平板机时的心电图。

冠状动脉造影检查是诊断冠心病最肯定的方法。

**十五、冠心病病人在冬春季节中要注意什么？**

气候变化可诱使冠心病病人发生急性心肌梗塞 阿尔卑斯地区在春秋季时 心肌梗塞的病人明显增多 我国山东地区在3～5 月份心肌梗塞的发病率最高；北京地区 每年的4月和11月是冠心病心肌梗塞的发病高峰期 秋末冬初和早春 我国多数地区的大气压 风速 温差都处于极不平衡状态 而变化多端的气候可能导致心脏血管发生痉挛 直接影响心脏本身的[血液供应；再则寒冷的季节里 常易发生](http://jck.39.net/jiancha/huaxue/bian/4e7d5.html)[感冒和支气管炎 这一切对患有冠心病的病人都十分不利 常是诱发心绞痛和心肌梗塞的主要诱因 因此 冠心病病人在冬春季节里应注意以下几个问题：](http://jbk.39.net/keshi/neike/huxi/48eea.html)

 (1)除坚持服用冠心病的常用药物外 还要备好保健盒 氧气等急救药品；

(2)如频繁发生心绞痛 要及时卧床休息 并及时到医院检查 治疗；

(3)坚持参加力所能及的体育锻炼 如户外散步 太极拳 气功等 但遇有骤冷 暴雪 大风等天气变化时 要留在室内活动 根据气温变化，及时更换衣服被褥 注意保暖；

(4)避免[疲劳紧张 情绪激动 尽量少参加社交活动和长途旅行 适当节制性生活；](http://zzk.39.net/zz/quanshen/503b3.html)

 (5)提倡用温水擦澡 以提高皮肤的抗寒能力 同时要积极防治感冒 气管炎等上呼吸道感染。

**十六、冠心病病人怎样选择食物？**

冠心病病人在选择食物时，应注意选择一些脂肪和胆固醇含量较低，而维生素、食物纤维、有益的无机盐和微量元素较多的，并有降血脂、抗凝血作用的食物。具体可从以下几类食物来选择。

1．可以随意进食的食物 ①各种谷类，尤其是粗粮。②豆类制品。③蔬菜，如洋葱、大蒜、金花菜、绿豆芽、扁豆等。④菌藻类，如香菇、木耳、海带、紫菜等。⑤各种瓜类、水果及茶叶。

2．适当进食的食物 ①瘦肉，包括瘦的猪肉、牛肉和家禽肉（去皮）。②鱼类，包括多数河鱼和海鱼。③植物油，包括豆油、玉米油、香油、花生油、鱼油、橄榄油。④奶类，包括去脂乳及其制品。⑤鸡蛋，包括蛋清、全蛋（每周2~3个）。

3．少食或忌食食物 ①动物脂肪，如猪油、黄油、羊油、鸡油等。②肥肉，包括猪、牛、羊等肥肉。③脑、骨髓、内脏、蛋黄、鱼子。④软体动物及贝壳类动物。⑤糖、酒、烟、巧克力等。

**十七、冠心病病人为何应少吃食盐？**

大量研究表明：高血压是冠心病的危险因素之一。调查资料发现，有相当比例的冠心病病人患有高血压，而高血压又有促进冠心病发展的作用。因此，控制高血压并设法降低血压水平，对冠心病的防治具有重要意义。同时，钠促进血液循环，增加心排血量，直接增加心脏负担，对心脏血流供应不足的冠心病人是不利的。目前普遍认为，钠摄入量在促进高血压发病中起着一定的作用。流行病学资料表明，食盐每日摄入高达20克的日本北部人，高血压发病率可高达40%，明显高于食盐每日摄入约5克的北美爱斯基摩人的发病率。研究还证明：平均每天少摄入5克食盐，平均舒张压可降低0.53千帕（4毫米汞柱）。因此，对已患有高血压的病人，限制食盐可作为一种非药物性治疗手段。

那么，冠心病病人应限制多少食盐为宜呢, 这要根据病人是否同时患有高血压，以及高血压的病情来决定。有人提出较为严格的限盐量，规定每日不超过5克。有的人较难做到并坚持。笔者认为，应根据自己的情况，逐渐限制食盐用量，使自己的口味渐渐习惯于低盐膳食。具体做法是：烹调时在菜肴出锅前将盐撒在食物上，盐味便可以明显地感觉出来；还可利用糖、醋、香料等调味品，来增加食物味道，以减少食盐用量。目前市场上出售的低钠盐，也是限盐（限钠）的一个较好的选择。

最后应指出，钾盐是可以保护心肌细胞的。促进钠排泄的降压药，常常增加钾排泄，造成体内缺钾。因此，膳食中于限盐（限钠）的同时，应多吃含钾的食物，例如五谷杂粮、豆类、肉类、蔬菜和水果均含有一定量的钾。动物性食品虽大多含钾比蔬菜、水果高，但钠含量、胆固醇含量较高，而蔬菜、水果含的钠极少，所以，应多吃水果、蔬菜来补钾。含钾高的食物有菠菜、萝卜、卷心菜、芹菜茎、南瓜、鲜豌豆、柠檬等，均可选食。

**十八、冠心病病人应如何对待吸烟和喝酒、咖啡及茶？**

1．吸烟： 目前，世界范围内大规模的研究表明，吸烟危险人体健康，对冠心病病人则更是有百害而无一利，有此恶习者要坚决戒掉。烟内所含的尼古丁可刺激血管发生痉挛，同时可使血压升高，心跳加快，诱发心绞痛。据报道，一位41岁男性，在连续吸烟20支后，发生急性心肌梗死死亡。国外也有研究发现，吸1支烟可减寿12分钟。吸烟既是公害，又是私害，应予禁绝。

2．喝酒： 大量喝酒可刺激脂肪组织分解，形成大量的脂肪酸，使肝脏合成的前β-脂蛋白量急剧增高。同时前β-脂蛋白和乳糜微粒在血中分解速度减慢而加重脂蛋白血症。以前有研究发现，喝酒可诱发心绞痛及心肌梗死。但80年代末期的研究认为，少量喝酒，尤其是低度酒，对心脏具有保护作用。因而美国心脏病协会推荐，冠心病病人即使患有心肌梗死，也可饮低度酒，喝酒量以1天不超过50克为宜。

3．喝咖啡： 咖啡中所含的咖啡因，可刺激血脂及血糖增高。1杯咖啡中约含咖啡因100毫克～150毫克。有研究发现，长期习惯于喝咖啡者如1天喝2杯以上，其血胆固醇水平及冠心病发病率，比不喝咖啡或每天喝1杯以下者明显增高。另外，喝咖啡可使体重增加。即使喝咖啡量很小，也可引起血胆固醇百分比比例失调。因而主张，高胆固醇血症者最好立即停喝咖啡。即使血胆固醇水平正常的健康人，也应尽可能地将咖啡摄入量降到最低限度。需要提醒大家的是，不管咖啡味道多好，都不如饮白开水对身体有利。

4．喝茶： 茶叶中所含的茶碱、维生素C和鞣酸对身体有益。我国按传统制作的茶叶，所含咖啡因 甚少，有助于消化，并有利尿作用。因此，只要不影响睡眠，一般是可以喝茶的。但也应尽量避免喝浓茶。

**十九、老年冠心病病人能否拔牙？**

一般说来，患有心脏病的老人，只要没有心力衰竭及严重的心律失常，都可以拔除坏牙。但是拔牙时，必须做好以下的心脏保护工作：

1．有冠心病心绞痛的病人，应先由内科治疗，病情稳定后再拔牙。拔牙前可服长效硝酸甘油片，同时身边要备有抗心绞痛的药物。必要时，口腔科医生和心脏科医生密切合作，并在心电监护下进行拔牙术。

2．拔牙时，麻醉剂最好选择利多卡因，尽量不要加入肾上腺素，以免出现心动过速而诱发心律失常或心力衰竭。

3．麻醉要安全，操作要熟练，动作要轻巧，尽量减少疼痛刺激、出血和损伤，以免引起患者精神紧张和血压的波动，从而增加心流域的负担。

4．如无特殊情况，应分期分批拔除坏牙。拔牙前后，应予抗感染预防处理。因为老年心脏病病人，抵抗力较正常人明显降低，拔牙形成的创面易发生感染。如无特殊情况，可口服抗生素。

**二十、有心肌梗死的病人为何不要饱餐和饱餐后沐浴？**

我们曾不止一次地听到和看到，有的心肌梗死病人，由于经不住家人或亲友的"好心"相劝和美味佳肴的引诱，多吃了几口就再次诱发了心肌梗死甚至死亡。还有的病人饱餐后即去沐浴，结果洗浴不久便倒于浴室，虽经积极抢救也无济于事。

这是为什么呢？在正常情况下，胃肠道的血管极其丰富，进食后，因消化与吸收的需要，心输出量增加，腹腔脏器处于充血状态。急性心肌梗死病人坏死的心肌没有收缩力，心功能很不好，在此基础上如果饱餐，一方面心脏输出量增加可加重心脏负荷；另一方面过饱使胃膨胀，横膈上移，进一步影响心脏功能；同时还可引起胃冠状反射，使冠状动脉收缩，血供减少，心肌进一步缺血、缺氧 而加 重心肌的功能不全；更有甚者，因饱餐后迷走神经兴奋而致窦房结节律性减低，可引起心跳停止。

基于以上原因，在饱餐后沐浴危险性就更大了。因为入浴后全身小血管扩张，使心脏和脑部更加缺血和缺氧，所以极易促成猝死。

**二十一、冠心病病人在运动中要注意什么？**

运动固然对冠心病病人有好处，但运动不当，给冠心病病人带来的危害也屡见不鲜。因此，冠心病病人在参加体育运动时，必须注意以下问题：

 1．运动前后避免情绪激动。精神紧张，情绪激动均可使血中儿茶酚胺增加，降低心室颤动阈。加上运动可有诱发室颤的危险，因此，对于心绞痛发作3天之内，心肌梗死后半年之内的病人，不宜做比较剧烈的运动。

2．运动前不宜饱餐。因为进食后人体内血液体供应需重新分配，流至胃肠帮助消化的血量增加，而心脏供血相对减少，易引起冠状动脉相对供血不足，从而发生心绞痛。

3．运动要循序渐进，持之以恒，平时不运动者，不要突然从事剧烈的运动。

4．运动时应避免穿得太厚，影响散热，增加心率。心率增快会使心肌耗氧量增加。

5．运动后避免马上洗热水澡。因为全身浸在热水中，必然造成广泛的血管扩张，使心脏供血相对减少。

6．运动后避免吸烟。有些人常把吸烟作为运动后的一种休息，这是十分有害的。因为运动后心脏有一个运动后易损期，吸烟易使血中游离脂肪酸上升和释放儿茶酚胺，加上尼古丁的作用而易诱发心脏意外。

**二十二、老年冠心病患者如何康复锻炼？**

老年人要听从医生的嘱咐 适当活动

运动量宜从轻量级开始 如轮替活动肢体 屈膝 摆动双臂 活动颈 肩关节 起坐 然后下床 躺在椅上 自己进餐 洗漱 入厕 逐渐增加活动量 以达到或接近梗死前的活动度为准。

步行是最方便的运动方式 多访友 作消遣活动 尽量避免奔跑 纵跃 因为有时会因此引起体位性低血压等不良反应。

太极拳也是冠心病患者的良好锻炼方式，高龄患者出汗反应差 因此散热也慢 故不耐热 所以在气温高时 或湿度高的情况下 应暂停运动锻炼

**二十三、冠心病病人外出旅游要注意些什么？**

冠心病病人外出旅游时，要注意以下几点：

1．旅游只限于心功能较好的病人 心功能Ⅱ级者，不可远游，尤其避免爬山、游泳等剧烈活动。心功能Ⅲ级者，只能在室内或住地周围的风景区进行活动。心肌梗死后康复期的患者，3个月内不能做长途旅游。

2．旅游前应到医院做一次全面检查 根据医生意见，确定自己能否长途旅游和旅游活动范围。旅游时要有人陪同并带有病情摘要、近期心电图和一般急救药，如硝酸甘油片、速效救心丸、异搏定、安定和地高辛等药。

3．避免过度疲劳 每日活动时间不超过6小时，睡眠休息时间不少于10小时。时间和日程安排宜松不宜紧，路途宜短不宜长，活动强度宜小不宜大。

4．带必要药品和有病及时就医 外出时胃肠炎和晕机、晕车是常易发生的，如不及时治疗，极易诱发心脏病。因此，要随身带上乘晕宁、安定和黄连素等药。一旦发病，应及早就医，切勿拖延，千万不可带病继续旅游，以免发生意外。

5．选择好的旅游季节 前苏联旅游科研机构的科学家们认为，春季旅游对神经系统、运动系统、内分泌系统尤其是心血管系统，有十分良好的影响，可以促进新陈代谢等生理变化过程。

**二十四、控制冠心病的关键是什么？**

控制冠心病的关键在于预防。虽然冠心病是中老年人的常见病和多发病，但其动脉粥样硬化的病理基础却始发于少儿期，这期间的几十年为预防工作提供了极为宝贵的机会。一级预防，防止冠状动脉粥样硬化的发生，消灭冠心病于萌芽状态；二级预防，提高全社区冠心病的早期检出率，加强治疗，防止病变发展并争取其逆转；三级预防，及时控制并发症，提高患者的生存质量，延长病人寿命。

冠心病的一级预防，即危险因素的干预。预防冠心病可采用针对全人群和高危人群两种预防策略。前者是通过改变某个人群、地区或国家与冠心病危险因素有关的生活行为习惯、社会结构和经济因素，以期降低人群中危险因素的均值；后者是针对具有１个或１个以上公认的（如高血压、吸烟等）与冠心病有明确因果关系的危险因素水平的降低，才能有效地减少冠心病的发生。目前公认冠心病危险因素包括男性、40岁以上的中老年人、有过早患冠心病的家族史、吸烟（现吸烟〉10支／日）、高血压、高血脂、重度肥胖（超重〉30％）、有明确的脑血管或周围血管阻塞的既往史。其中，高血压、高胆固醇及吸烟被认为是冠心病最主要的3个危险因素。除性别、年龄和家族史外，其它危险因素都可以预防和治疗。

冠心病病变始儿童，动脉粥样硬化病变的形成是一个漫长的过程，因此，必须从小养成良好的生活习惯、健康的生活方式。膳食结构要合理，避免摄入过多的脂肪和大量的甜食，加强体育锻炼，预防肥胖、高脂血症、高血压和糖尿病的发生。超重和肥胖者更应主动减少热量摄入，并加强运动量。高血压、高脂血症和糖尿病患者，除重视危险因素干预外，更要积极控制好血压、血糖和血脂。大力宣传戒烟活动，特别是要阻止儿童成为新一代烟民。

**二十五、预防冠心病的措施**

不吸烟

只食用少量的牛油 奶油及各种油腻食物

将你习惯食用的肉类量减少 将你食用的肉上脂肪除去 吃烧煮的肉 不要吃油煎的肉 每周最多只吃三只鸡蛋

吃大量水果及蔬菜 但饮食要维持平衡均匀

减少盐的摄食量 摄食盐量低可以降[低血压并且减少发展冠状动脉病的危险。](http://zzk.39.net/zz/xiongbu/4ff97.html)

经常运动 每周做两 三次剧烈运动 可减少得心脏疾病的危险 但由于突然做剧烈运动很危险 必须以渐进的方式来开始实行你的运动计划

应付精神压力 寻求各种途径来调解生活上的压力 可以培养嗜好或通过运动来松懈日常生活中的紧张情绪。

控制高血压 高胆固醇血症和糖尿病

定时检查身体并遵照医嘱

**二十六、治疗冠心病的常用药物有哪几类？**

治疗冠心病的药物很多，常用的主要有：

①硝酸酯制剂：主要包括硝酸甘油、消心痛、５－单硝酸山梨醇、长效硝酸甘油制剂等。

②肾上腺素能β受体阻滞剂（β阻滞剂）：常用的制剂有普萘洛尔、氧烯洛尔、烯丙洛尔、吲哚洛尔、美托洛尔、阿替洛尔、纳多洛尔。

③钙通道阻滞剂：常用制剂有维拉帕米、硝苯地平、地尔硫卓、尼卡地平等。

④抗血小板药物：如阿斯匹林、双嘧达莫、苯磺唑磺等。

⑤调整血脂药物：如烟酸、普伐他汀、洛伐他汀、辛伐他汀等。

⑥溶血栓药物：如华法令、肝素、尿激酶、链激酶等。

⑦中医中药：以活血（常用丹参、红花、川芎、蒲黄、郁金等）和化瘀（常用苏合香丸、苏冰滴丸、宽胸丸、保心丸、麝香保心丸等）最为常用。

**二十七、冠心病急性发作时的治疗**

1．心绞痛：应立即停止体力活动,就地休息,设法消除寒冷,情绪激动等诱因;立即舌下含化硝酸甘油或消心痛1片,如未缓解,隔5到10分钟再含化一次,连续3次含化无效,[胸痛持续15分钟以上者有发生心肌梗塞的可能,应立即送医院等急救场所;可口服安定3毫克,有条件者应吸氧10到30分钟 冠心病病人应随身携带硝酸甘油等药物,一旦出现胸痛立即含服,并注意不要使用失效的药物 稳定型心绞痛在休息和含化硝酸甘油后心绞痛会缓解,不稳定型心绞痛是一个严重而潜在危险的疾病,应立即送医院治疗和严密观察](http://zzk.39.net/zz/quanshen/5050a.html)

2．心肌梗塞：急性心肌梗塞死亡率高,其中半数以上病人是在住院前死亡的,大多数死亡发生在发病后1小时内,一般由心室纤颤引起 所以就地急救措施和迅速转送医院至关重要 在高危病人([高血压糖尿病,既往有心绞痛发作者)中一旦发生以下情况：胸部不适,极度](http://zzk.39.net/zz/xiongbu/500cf.html)[疲劳呼吸困难](http://zzk.39.net/zz/quanshen/503b3.html),尤其伴有大汗,头昏,[心悸濒死感时](http://zzk.39.net/zz/xiongbu/50027.html),要高度怀疑发生了心肌梗塞,应立即送距离最近的,有条件作心电图,心电监护,直流电除颤,静脉溶栓的医疗机构 同时保持镇静,不要引起病人的惊慌和恐惧,并含化硝酸甘油,或者速效救心丸,冠心舒合丸等,有条件可肌注罂粟碱,或杜冷丁,以及安定,并保持通风和吸氧,如无禁忌症,立即口服阿斯匹林300毫克 如发生室速,室颤等恶性心律失常立即予直流电除颤 一旦发生心脏骤停,应立即人工呼吸和胸外心脏按压进行心肺复苏。,,,

3．急性心衰和心源性[休克：急性心肌梗塞和缺血型](http://zzk.39.net/zz/quanshen/50223.html)[心肌病都可能发生急性心衰](http://jbk.39.net/keshi/neike/xxgnk/4cb30.html),由于大面积心肌坏死所致.多为急性左心衰,患者出现严重呼吸困难,伴烦躁不安,窒息感,面色青灰,口唇[紫绀大汗淋漓](http://zzk.39.net/zz/pifu/5025a.html),咳嗽,咯大量白色或粉红色泡沫痰,这种情况必须立即送医院抢救。,

**二十八、冠心病发作时怎样使用保健盒进行自救？**

冠心病病人常发病骤然，或有心绞痛，或有心律失常，甚至可发生心肌梗死，如果能及时用药，常可转危为安。但往往是冠心病病人在发病时身边无人，家又离医院很远，这时如果身边带有一个保健盒再好不过了。一般的保健盒，常分别装有硝酸甘油（速效）、长效硝酸甘油、潘生丁、亚硝酸异戊酯、安定、芬那露等几中药品。

通常选用其中3～5种药品装在小的铝盒或其它合适的药瓶中，平时带在身边，晚睡放在床边，用时随手可取。那么在什么情况下，如何应用这些药物呢？如果你突然发作了心绞痛，要立刻卧床休息，同时马上舌下含服速效硝酸甘油1～2片，一般用药后2～3分钟就可缓解，药效可维持20～30分钟，可重复用药2～3次。如心绞痛发作时，伴有精神紧张或烦躁不安，或伴有心动过速，可同时服用安定或芬那露1～2片，有助于解除你的焦虑，对缓解心绞痛大有好处。如近期反复发生心绞痛，除临时服用速效硝酸甘油外，还要坚持每日3次定时服用消心痛和潘生丁，剂量为消心痛10毫克/次，潘生丁50毫克/次。这两种药者是血管扩张药，对改善心肌血供，减少血小板凝集，减轻心脏负担，缓解心绞痛大有好处。

为了防止心绞痛发作，改善心肌供血，在劳累和情绪波动前，可服用10毫克的长效硝酸甘油。目前不太主张用亚硝酸异戊酯，主要是因它有迅速扩张血管而易引起冠状动脉"盗血"现象（使用某些扩血管药物时，向缺血区供血的血管发生病变时，对扩血管药不敏感，从而血管扩张程度很轻甚至不扩张；相反非缺血区供血的正常血管扩张程度较大，致使非缺血区血供增加而缺血区供血更趋减少的现象）。如果经过保健盒的药物治疗后，你的症状仍未缓解，则要考虑是否药物失效或治疗不对路，就应立即找医生看看，以免耽误病情，同时要提醒的是保健盒的药物要定期更换。

**二十九、冠心病的药物 介入 手术搭桥治疗**

药物只能控制症状 能稳定冠状动脉里的斑块 药物是基础 介入是最近20年发展起来的 2000年之后已经成熟起来 不仅仅是控制症状 而且有可能彻底根治 把窄的血管撑起来 介入简单 无创 不用开胸 而且立竿见影 技术上是高风险的 需要一定的培训 有一定的条件才能做 也需要病人的配合 支架一旦形成血栓那就要出问题了 有1%的发生率 总体来说 现在人们除了追求生活以外还追求生活质量 一个40岁的男性 天天出去跑 要打高尔夫 要爬山 天天吃药虽然不死但是觉得活的没什么意思 就要做介入 不能做介入的或者经济条件比较差的 介入要做七八个支架才能解决 那干脆放弃 考虑搭桥

实际上介入跟搭桥手术是一个东西 介入是把里面的堵塞撑开疏通 搭桥是开胸从上游弄一根管子到下游去 重新开辟一条通道 从理论上来说 药物 介入跟手术对低危的稳定病人效果一样 做介入也好 做搭桥也好 吃药也好 不会死人的 这是公认的研究结果 但是介入的治疗过程中间对病人的手术损伤要大大小于外科手术,康复起来的时间要短很多,一般说来外科和介入有不同的手术适应症 一般会考虑患者的经济适应能力和身体患病的具体情况。介入治疗的人可以爬山 但是吃药的人绝对不能爬山 搭桥的也可以爬 但是搭桥的人复发的少 介入的人复发的多 搭桥的人再狭窄的几率小 现在有药物支架 可预防复发 药物支架和搭桥的复发率估计差不多。

**三十、中医对冠心病的治疗手段或处方用药**

在治疗上 适宜根据患者的临床表现的不同而采用急则治标 缓则治本 先攻后补 先补后攻 攻补兼施等治疗手法 切不能一攻到底或只知补虚而忽视疏导痰瘀。

1.心阳亏虚：心阳不足为心气不足的重症 古人说：“心包相火附于命门 ”心阳不足的根源乃是命门火衰 立方以附子(用量为10g左右 本品有大毒 不可多用) 桂枝益肾中真火 《名医方论》说：“火少则生气 火壮则食气” 故不用肉桂而用桂枝 且附 桂用量不大 其意思在于微微生火 以防过燥伤阴 补气之品多用红参(最好是高丽参或吉林参) 冠心病人都有不同程度的心脉瘀阻 所以多配以红花 桃仁 丹参等活血化瘀药 在活血化瘀的同时 还应注意理气 行气 意在调整脏腑的切机以调理气血 去瘀生新 冠心病人的血瘀既可因寒而凝 也可因热而结 寒凝瘀久且可化热 凡热结而瘀或瘀久化热者 如仍用温通之品 必然助热伤阴 而应凉血化瘀 其意在于护阴 此外 冠心病患者亦有不同程度上的痰浊阻滞 故往往采用芳香化浊 温化痰饮的法半夏 陈皮 天南星 蔻仁 春砂仁 但如蕴久化热者仍用温化之品 必助热伤阴 此时必须清化

2.心阴虚损：冠心病者多见于老人 而老人常见素体阴虚 在病变过程中 多易出现阴虚尤其是肾阴虚的症候 久之阴损气伤 或气虚津液生成不足 都可出现气阴两虚的症候 因而适宜采用益气养阴的方法治疗 五味子 太子参 白芍 麦冬 石斛均为常用益气养阴之品 虽药味不多 而兼有补气 清热 滋阴 敛阳四法 养阴能增加气血的来源 益气能行血 故益气养阴的同时 又能促进活血化瘀的作用 太子参 五味子 麦冬为生脉散 根据现代药理研究 表明生脉散有稳定而持久的强心作用 对已经被损伤的冠状动脉内壁有修补作用 同时还能稳定正常的血压

**三十一、冠心病心肝失调型药膳**

 冠心病心肝失调的主要症状是心前区绞痛，心悸，气短，情志不舒，失眠多梦等证。能治疗冠心病心肝失调的中药有：川芎、天麻、酸枣仁、茯神、桑寄生、知母、炙甘草、菊花等。能制作的食物有猪肉、牛肉、羊肉、禽蛋、蔬菜、水果、海鲜及滋补心肝的一切食物。

天麻蒸鸡蛋

配方：天麻10克 鸡蛋1枚 盐5克 芝麻油5克 酱油10克 葱花5克

制作：1.把鸡蛋打入蒸盆内，葱切花，天麻烘干打成细粉。

2.把葱花、天麻粉、盐、芝麻油放入鸡蛋蒸盆内，拌匀，加适量清水。

3.把蒸盆置武火大气蒸笼内蒸15分钟即成。

食法：每日1次，佐餐食用。

功效：补养肝肾，养心安神

妙香舌片

配方：酸枣仁12克 猪舌1只 冬菇30克 葱10克 黑木耳20克 酱油10克 盐5克 绍酒10克 生粉20克 姜5克 素油50克

制作：1.把酸枣仁烘干，研成细粉，猪舌洗净，用沸水焯透，刮去外层皮肤，切簿片。黑木耳洗净，发透，去蒂根，撒成瓣状。葱切段，姜切丝。

2.把猪舌放碗中，加入酸枣仁粉，绍酒，盐，酱油，生粉，姜，葱各一半，加适量水调稠状，待用。

3.把炒勺放在中火上烧热，加入素油烧六成熟时，下入姜、葱另一半爆香，再下入腌渍之舌片，翻炒2分钟，下入黑木耳，炒熟即成。

食法：每日1次，每次吃猪舌50克，吃黑木耳。

功效：滋补肝肾，宁心安神。心肝失调，心悸多梦冠心病患者食用。

甘菊饮

配方：菊花10克 甘草3克 白糖30克

制作：1.把菊花洗净，去杂质，甘草洗净，切薄片。

2.把菊花、甘草放入锅内，加水30毫升。把锅置中火烧沸，再用文火煮15分钟，过滤，除去药渣，留汁。

3.在药汁内加入白糖拌匀即成。

食法：代茶饮用 功效：滋补心肝，理气明目。心肝失调之冠心病患者饮用。

妙香茯神汤

配方：酸枣仁12克 茯神10克 猪瘦肉50克 鸡蛋1只 生粉20克 菜胆100克 酱油10克 葱10克 姜5克 盐5克 生素油50克

制作：1.把茯神、酸枣仁去杂质，猪瘦肉洗净，切片，菜胆洗净，切4厘米段，葱切花，姜切丝。

2.把茯神、酸枣仁放炖杯内，加水50毫升，用中火煎者25分钟，去渣，留汁待用。

3.把猪肉放入碗内，加入生粉、药汁、鸡蛋、盐、酱油、姜、葱，拌成稠状待用。

4.把锅置武火上烧热，加入素油，烧六成熟时，下入姜、葱爆香，加入上汤600毫升，烧沸，投入猪肉煮熟，加入菜胆，断生即成。

食法：每日1次，佐餐食用。

功效：滋补气血，宁心安神，行气疏肝，心肝失调多梦之冠心病者食用。

陈皮参芪煲猪心

配方：陈皮3克 党参15克 黄芪15克 猪心1只 绍酒适量 盐5克 胡萝卜100克 生素油30克

制作：1.把陈皮、党参、黄芪洗净，陈皮切3厘米见方的块，党参切片，黄芪切片，胡萝卜切4厘米见方的块，猪心洗净，切成3厘米见方的块。

2.把锅置中火上烧热，加入素油，六成熟时，加入猪心、胡萝卜、绍酒、盐、党参、陈皮、黄芪，加鸡汤300毫升，烧沸，再用文火煲至浓稠即成。

食法：每日1次，每次食猪心30克，胡萝卜50克，木耳随意食用。

功效：补心气，益气血，疏肝解郁。心肝失调之冠心病患者食用。

人参麦冬炖瘦肉

配方：人参10克 麦冬10克 五味子6克 瘦猪肉50克 冬菇30克 姜5克 葱10克 盐5克

制作：1.把人参洗净、润透、切片，麦冬洗净去心，五味子洗净。冬菇洗净，一切两半，姜拍松，葱切段。猪肉切4厘米见方的块。

2.把瘦猪肉放入炖锅内，加入冬菇、姜、葱、盐、人参、麦冬、五味子，注入鸡汤或上汤600毫升。 3.把炖锅置武火上烧沸，再用文火煮1小时即成。

食法：每日1次，佐餐食用。

功效：活血清热，滋阴养心。心阴虚之冠心病患者食用。

人参灵芝煲兔肉

配方：人参10克 麦冬10克 兔肉100克 绍酒10克 盐5克 葱10克 姜5克 生素油30克

制作：1.人参润透切片，灵芝润透切片，兔肉洗净，切3厘米见方的块，葱切段，姜拍松。

2.把人参、灵芝、兔肉放入碗内，加入绍酒、盐拌匀，淹渍30分钟。

3.把锅置中火上，加入素油烧，六成熟时，下入兔肉，加上汤400毫升，加入人参、灵芝片、姜、葱，用武火烧沸，文火煲25分钟即成。

食法：每日1次，每次食兔肉30克，吃人参(灵芝可不吃弃去)。 功效：滋阴养心，补益气血，疏肝行气。心肝失调之冠心病患者食用。

天麻首乌炒肝心

配方：天麻10克 何首乌15克 猪肝50克 猪心50克 绍酒10克 鸡蛋1只 葱10克 姜5克 鸡汤100克 生粉20克 素油50克 花菜100克

制作：1.把天麻、首乌烘干，研成细粉；猪肝洗净切片；猪心一切两半，除去血管，切片；花菜洗净，撕成大朵花；姜切丝，葱切段。

2.把猪心片、肝片放入碗内，加入生粉，打入鸡蛋，一半姜、葱注入少许鸡汤拌匀，待用。

3.把炒勺置武火上，加入素油，烧六成熟时，下入葱、姜爆香，下入肝、心片，加入首乌、天麻粉，炒匀，然后加入花菜和剩下鸡汤，待花菜熟透即成。

食法：每日1次，每次吃肝、心30克，随意食花菜。

功效：滋补肝心，宁心安神。心肝虚损之心脏病患者食用。

百合玉竹粥

配方：百合20克 玉竹20克 大米100克

制作：1.把百合洗净，撕成瓣状，玉竹切成4厘米段，大米淘洗干净。

2.把百合、玉竹放入锅内，加入大米，水100毫升。

3.把锅置武火上烧沸，用文火煮45分钟即成。

食法：每日1次，当早餐食用。

功效：滋阴润燥，生津止渴。心肝失调之冠心病患者食用。


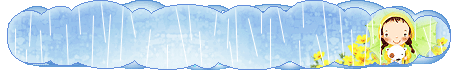
 **冠心病出院后的健康教育指导**

患者康复出院后要继续吃什么药、定期做什么检查听医生的，在护理方面提点建议供参考：

***一、保养***

**1 .注意气候变化**

冬春季节是各种疾病发生和流行的季节，冬季早晚温差大，春天时风时雨的天气，均易诱发心绞痛，因此，病人应随天气变化及时增减衣物，冷天注意保暖避免冷风刺激。

**2.建立良好的生活方式**

生活行为方式是影响冠心病的发生、发展及转归的重要因素之一，高血压、高血脂、是冠心病的危险因素，注意生活规律，控制情绪，放松精神，愉快生活，保持心情平和，保证睡眠质量，不吸烟，不饮浓茶、咖啡，养成定时排便的习惯，保持大便通畅，避免便秘，以免用力排便而加重病情，适当锻炼，避免做不胜任的体力劳动。

**3. 坚持服药**

高血压、冠心病患者需要长期服药。遵医嘱不可随意停用或增减，在用药过程中，学会自我监测，如使用抗凝药阿司匹林时，应饭后服用，出现牙龈出血、呕吐、黑便和皮肤出血点时立即停药；使用利尿药，注意观察尿量，出现异常随时就诊。外出时要随身携带硝酸甘油，居家时硝酸甘油放在易取之处，定位放置，家人也应知道，以便发病时及时取用;β受体阻滞剂与钙通道阻滞剂合用会出现抑制心脏的危险，学会自测脉搏，脉搏<60次/min，应该暂停服药，去医院就诊。

**4.警惕不明原因的疼痛**

冠心病以心绞痛型、心肌梗死型最常见。心绞痛常表现为胸骨中上部的压榨痛、紧缩感、窒息感及胸痛逐渐加重，且数分钟达到高潮等症状;心肌梗死是冠状动脉血供急剧减少或中断所导致，使相应的心肌严重持久地急性缺血。

发现此情况赶急服硝酸甘油和拨120往医院治疗。

***二、忌吃***

忌吃油炸食品、鱼子、蛋黄、肥肉等高醇高脂食品，控制含糖分高和咸的食物。

***三、多吃***

对冠心病病人来说，限盐勿忘补钾。多吃富含钾元素食物，如豆类及其制品、马铃薯、紫菜、海带、香菇、蘑菇、山药、春笋、冬笋、木耳、荞麦、以及香蕉、西瓜等。以利缓和钠，保护心肌细胞。多吃能降血脂食物如牛奶、羊奶、黄豆、赤小豆、绿豆、蚕豆、豌豆、扁豆、芸豆、豆芽、胡萝卜、菜花、韭菜、大蒜、大葱、洋葱、生姜、番茄、鲜菇、紫菜、鱼类、柑桔、苹果、山楂等。

***四、食疗便方：***

1.海带9克，草决明15克水煎（除去药渣），同煮，吃海带饮汤，或常以海带煎水，吃海带饮汤。此方兼治肥胖症。

2.菊花3克，山楂（切片）、草决明各15克，放入保温杯中用沸水冲泡，盖严，浸半小时，代茶饮。

3.大枣、芹菜根煎汤，经常适量服用，可兼治高胆固醇。
