# Supplementary material for: The effects of Guozhuang dance on exercise self-efficacy in coronary heart disease patients following percutaneous coronary intervention: a randomized controlled trial
Source: Front Cardiovasc Med. 2026 Jan 19;12:1688894. doi: 10.3389/fcvm.2025.1688894 (PMC12862062; doi:10.3389/fcvm.2025.1688894)
Supplement: Supplementary Data Sheet 4 — Per-protocol analysis. [file Datasheet4.docx]

# **Per**-**protocol** **analysis**

A separate per-protocol (PP) analysis comparing only the 81 completers (intervention n = 40; control n = 41). who:

1.Completed all four assessment time points (T0, Tl, T2, and T3)

2.Had no missing data for primary or secondary outcome measures

3. Adhered to the intervention protocol (for intervention group participants)

Exclusions from PP Analysis:

- 5 participants withdrew due to inability to complete the exercise protocol (3 from intervention group, 2 from control group)
- 2 participants were lost to follow-up (1 from each group)
- Total attrition:7 participants (7.95%)

####
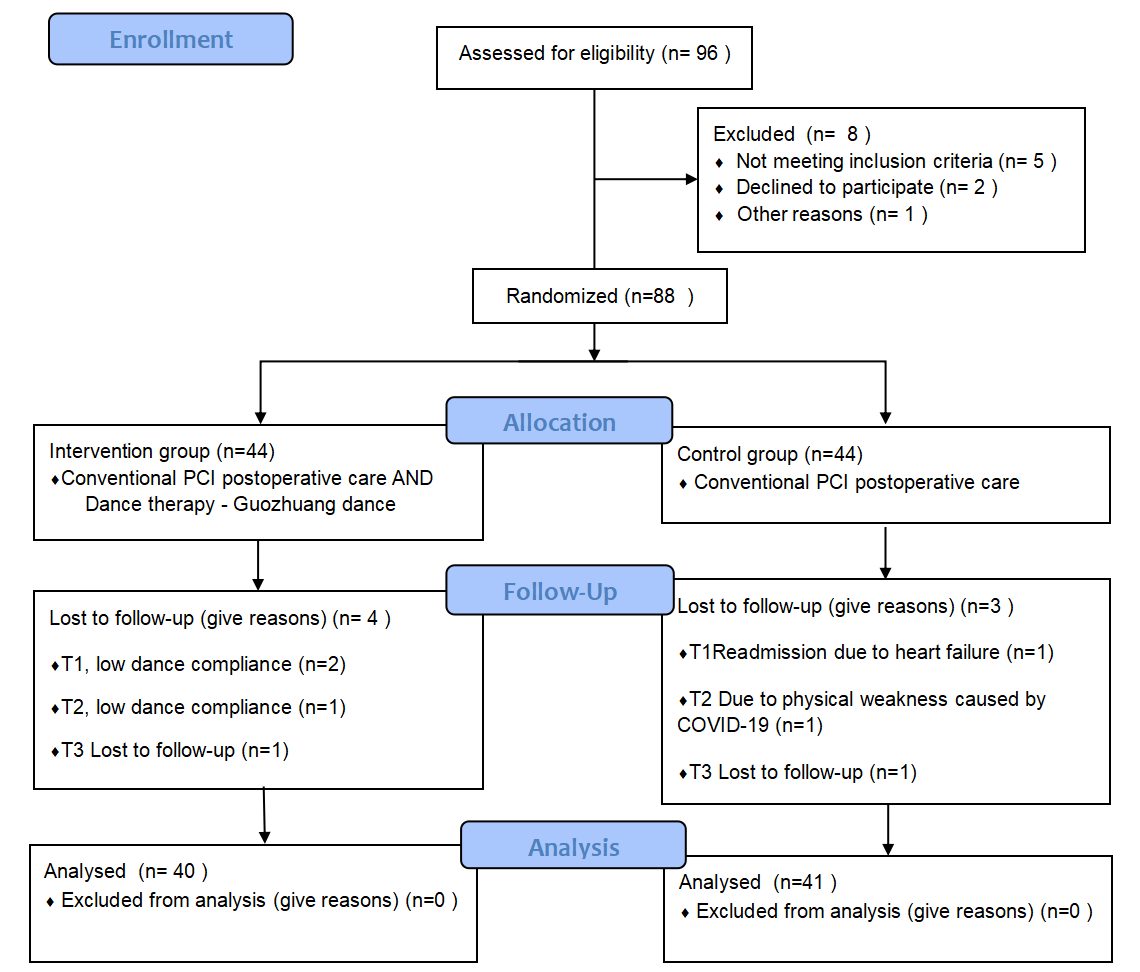


Figure 1. Participant Attrition Flowchart

**3. Results**

**3.1 General Characteristics of the Study Subjects in the Two Groups**

**3.1.1 Comparison of General Characteristics Between the Two Groups**

A total of 88 patients were enrolled in this study, with 44 patients in the intervention group and 44 in the control group. The mean age of patients in the intervention group was 62.59 ± 6.70 years, while that in the control group was 61.52 ± 8.15 years. Among them, 29 patients (65.91%) in the intervention group and 33 patients (75.00%) in the control group were male. Regarding occupational status, 52.27% of participants were retired or on medical leave, while 15.91%, 9.10%, and 22.72% were unemployed, part-time employed, and fully employed, respectively. In the intervention group, 22.73% of patients reported exercising 3 to 7 times per week, whereas 40.91% reported almost no physical activity. Furthermore, 56.82% of patients exercised for less than 30 minutes per session, and only 4.54% exercised for more than 60 minutes per session. Concerning post-PCI stent implantation, 67.05% of patients received a single stent. There were no statistically significant differences in baseline demographic or clinical characteristics between the two groups (*P* > 0.05), indicating comparability and balance. Detailed information is presented in Table 1.

**3.1.2 Participant Attrition**

During the course of the study, five participants withdrew due to their inability to complete the exercise protocol—three from the intervention group and two from the control group. Additionally, two participants were lost to follow-up, one from each group. Details are presented in Figure 1. A total of 81 participants completed the study, including 40 in the intervention group and 41 in the control group, resulting in an overall attrition rate of 7.95%.

**3.2 Scale Scores at Each Time Point in Both Groups**

The scale scores at each time point for both groups are summarized in Table 2. After 12 weeks of intervention, the intervention group had a significantly higher MSES score (60.56 ± 11.01) compared to the control group (40.20 ± 8.52), with a statistically significant difference (*P*< 0.05). The quality of life score in the intervention group was 80.18 ± 9.38, higher than that in the control group (74.49 ± 7.02), also showing a statistically significant difference (*P* < 0.05). The intervention group achieved significantly greater 6MWD (447.63±31.02 m) compared to the control group (424.95±28.96 m, *P* < 0.05). Physical activity scores were also significantly higher in the intervention group [1860.00 (1608.00, 2161.00)] versus the control group [1358.00 (1092.00, 1641.50), *P* < 0.05]. Significant between-group differences were

observed for all scale scores after the 12-week intervention.

**3.3 Comparison of Exercise Self-Efficacy Scores Between the Two Groups**

**3.3.1 Comparison of Exercise Self-Efficacy Scores at Different Time Points**

(a) To explore the effect of the intervention on exercise self-efficacy (ESE) in both groups, a generalized estimating equation (GEE) was used to analyze the repeated measures data. The results showed a significant interaction effect between time and group for the MSES score (*P* < 0.001), as shown in Table 3. This indicates that Guozhuang dance had differential effects on MSES scores across different time points. The trend of score changes over time is illustrated in Figure 3. Further analysis of the main effects of time and group is warranted.

(b) Main Effect of Time: Using T0 as a covariate to adjust for baseline effects, the results showed that, in the control group, MSES scores at T2 and T3 did not differ significantly from those at T1 (P > 0.05). In contrast, in the intervention group, MSES scores at both T2 and T3 were significantly higher than at T1 (P < 0.001), as shown in Table 4.

(c) Main Effect of Group: Using T0 as a covariate to adjust for baseline effects, the results showed that, at both T2 and T3, the MSES scores in the intervention group were significantly higher than those in the control group (P < 0.001). Furthermore, the mean difference between the two groups increased progressively over time, as shown in Table 5.

**3.3.2 Comparison of MSES Dimensional Scores Between the Two Groups**

At week 12, the intervention group had a total ESE score of 60.56 ± 11.01, while the control group scored 40.20 ± 8.52. Within the intervention group, the task completion efficacy score was 24.53 ± 4.78, the problem-solving efficacy score was 13.60 ± 3.71, and the time management efficacy score was 23.68 ± 4.88. These results indicate that problem-solving efficacy was the lowest among the three dimensions in both groups, as detailed in Table 6. Statistically significant differences were observed between the two groups across all dimensions (*P*< 0.001), as illustrated in in Figure 4.

**3.4 Comparison of 6MWD at Different Time Points Between the Two Groups**

(a) Generalized estimating equation analysis revealed significant differences in 6-minute walking distance (6MWD) between the intervention and control groups at different time points (*P*< 0.001). A significant interaction effect was also observed between time and intervention (*P* < 0.001), as shown in Table 7. These findings suggest that Guozhuang dance had varying effects on 6MWD over time, as illustrated in Figure 5, warranting further analysis of the main effects of time and group.

(b) Main Effect of Time: Using T0 as a covariate to adjust for baseline effects, the results showed that in the control group, 6MWD at all subsequent time points did not differ significantly from T1 (*P* > 0.05). In contrast, in the intervention group, 6MWD at T2 and T3 was significantly higher than at T1 (*P* < 0.001). Moreover, the mean difference between the two groups increased progressively over time, as shown in Table 8.

(c) Main Effect of Group: Using T0 as a covariate to adjust for baseline effects, the results showed that, compared to the control group, the intervention group exhibited significantly higher 6MWD at both T2 and T3 (*P* < 0.001), as shown in Table 9.

3.5 Comparison of Physical Activity at Different Time Points Between the Two Groups

(a) GEE analysis showed that IPAQ scores differed significantly between the control and intervention groups at different time points (*P* < 0.001). However, no significant interaction effect was found between time and intervention (*P* = 0.409 > 0.05), indicating a focus on the main effects. Details are presented in Table 10, and the trends in physical activity over time are illustrated in Figure 6.

(b) Comparison of Main Effects: Using T0 as a covariate to adjust for baseline effects, the results showed a significant main effect of time on physical activity scores, with a Wald χ² value of 7.529 (*P* < 0.05). A significant main effect of group was also observed for IPAQ scores (*P* < 0.05). These findings indicate that physical activity levels in post-PCI patients varied across different time points, and that the Guozhuang dance intervention had a statistically significant effect on improving physical activity.

Using T1 as the reference point, a significant difference in physical activity was observed at T2 (*P* < 0.05). Compared to the control group, the intervention group also showed significantly higher physical activity levels (*P* < 0.05), as presented in Table 11.

**3.6 Comparison of Quality of Life at Different Time Points Between the Two Groups**

(a) With the extension of the intervention period, quality of life improved in both groups. However, the degree of improvement was consistently greater in the intervention group compared to the control group across all time points, with the difference being statistically significant (*P* < 0.001). No significant interaction effect was found between time and intervention (*P* = 0.196 > 0.05), indicating that the main effects should be the focus. Detailed results are shown in Table 12, and the trend in quality of life over time is illustrated in Figure 7.

(b) Comparison of Main Effects: Using T0 as a covariate to adjust for baseline effects, the results showed a significant main effect of time on quality of life scores (CQQC), with a Wald χ² value of 689.115 (*P* < 0.05). A significant main effect of group was also observed (*P* < 0.05), indicating that quality of life levels in post-PCI patients differed across time points. The Guozhuang dance intervention had a statistically significant impact on quality of life improvement. Using T1 as the reference, a significant difference in quality of life was found at T2 (*P* < 0.05). Compared to the control group, the intervention group demonstrated a statistically significant improvement in quality of life (*P* < 0.05), as shown in Table 13.

Table 1 Comparison of General Characteristics Between Two Groups

| Characteristics | Intervention Group (n=44) | Control Group (n=44) | Test Statistic | *P* valve |
| --- | --- | --- | --- | --- |
| Age (years, x̄ ± s) | 62.59±6.70 | 61.52±8.15 | 0.671^1)^ | 0.504 |
| Gender |  |  | 0.873^2)^ | 0.350 |
| Male | 29（65.91） | 33（75.00） |  |  |
| Female | 15（34.09） | 11（25.00） |  |  |
| Education Level |  |  | 2.377^3)^ | 0.526 |
| Primary school or below | 14（31.82） | 20（45.45） |  |  |
| Junior high school | 17（38.64） | 12（27.27） |  |  |
| High school | 11（25.00） | 9（20.45） |  |  |
| College or above | 2（4.54） | 3（6.83） |  |  |
| Marital Status |  |  | 0.000^3)^ | 1.000 |
| Married | 40（90.90） | 39（88.64） |  |  |
| Single | 4（9.10） | 5（11.36） |  |  |
| Current Work Status |  |  | 3.025^2)^ | 0.388 |
| Unemployed | 7（15.91） | 7（15.91） |  |  |
| Retired or sick leave | 23（52.27） | 21（47.73） |  |  |
| Part-time | 4（9.10） | 1（2.27） |  |  |
| Full-time | 10（22.72） | 15（34.09） |  |  |
| Household Monthly Income (CNY) |  |  | 1.492^3)^ | 0.474 |
| < 2000 | 1（2.27） | 0（0） |  |  |
| 2000–4000 | 12（27.27） | 11（25.00） |  |  |
| > 4000 | 31（70.46） | 33（75.00） |  |  |
| Living Arrangement |  |  | 4.598^2)^ | 0.100 |
| Nursing home | 0（0） | 2（4.55） |  |  |
| Living with spouse | 34（77.27） | 37（84.09） |  |  |
| Living with children/friends | 10（22.73） | 5（11.36） |  |  |
| Medical Insurance Type |  |  | 3.567^3)^ | 0.168 |
| Full out-of-pocket | 20（45.45） | 18（40.91） |  |  |
| Insurance reimbursement | 23（52.27） | 21（47.73） |  |  |
| Rural cooperative | 1（2.27） | 5（11.36） |  |  |
| Post-PCI Time |  |  | 0.723³) | 0.395 |
| ≤1 year | 38 (86.36%) | 35 (79.55%) |  |  |
| >1 year | 6 (13.64%) | 9 (20.45%) |  |  |
| Family History of CHD |  |  | 0.180^2)^ | 0.914 |
| Yes | 3（6.82） | 4（9.09） |  |  |
| No | 41 (93.18%) | 40 (90.91%) |  |  |
| Exercise Types |  |  | 0.088²) | 0.767 |
| One type | 14（31.82） | 13（29.55） |  |  |
| Two or more types | 27（61.36） | 27（61.36） |  |  |
| Exercise Frequency |  |  | 2.668^3)^ | 0.102 |
| Frequent (2–3/week) | 10（22.73） | 15（34.09） |  |  |
| Occasional (1–2/month) | 16 (36.36%) | 16 (40.91%) |  |  |
| Rare or none (<2/month) | 18 (40.91%) | 11 (25.00%) |  |  |
| Exercise Duration |  |  | 1.396^3)^ | 0.514 |
| <30 min/session | 25（56.82） | 30（68.18） |  |  |
| 30–60 min/session | 17（38.64） | 13（29.55） |  |  |
| >60 min/session | 2（4.54） | 1（2.27） |  |  |
| Angiographic Report |  |  | -1.550^3)^ | 0.121 |
| Single-vessel disease | 21（47.73） | 13（29.55） |  |  |
| Double-vessel disease | 20（45.45） | 28（63.63） |  |  |
| Triple-vessel or above | 3（6.82） | 3（6.82） |  |  |
| Number of Stents |  |  | 1.286^2)^ | 0.257 |
| One | 32（72.73） | 27（61.36） |  |  |
| Two | 12（27.27） | 17（38.64） |  |  |

1)t-value 2) X2-value 3) Z-value

Table 2. Scale Scores of Patients in Both Groups at Each Time Point

| Variable |  | MSES | CQQC | 6MWD | IPAQ |
| --- | --- | --- | --- | --- | --- |
| T0 | 1 | 38.65±10.18 | 56.93±8.95 | 401.20±41.88 | 603.00（432.00，788.00） |
|  | 0 | 39.80±8.31 | 55.73±8.82 | 415.76±34.89 | 566.00（432.00，723.00） |
|  | *t/Z* | 0.559 | 0.605 | -1.701 | -0.799 |
|  | *p* | 0.577 | 0.547 | 0.093 | 0.424 |
| T1 | 1 | 51.78±10.32 | 70.05±9.18 | 437.23±36.50 | 1449.00（1177.00，2094.00） |
|  | 0 | 40.02±8.61 | 65.76±7.05 | 427.24±31.96 | 1100.00（861.00，1428.00） |
|  | *t/Z* | -5.569 | 2.355 | 1.309 | -3.554 |
|  | *p* | ＜0.001 | 0.021 | 0.194 | ＜0.001 |
| T2 | 1 | 60.56±11.01 | 80.18±9.38 | 447.63±31.02 | 1860.00（1608.00，2161.00） |
|  | 0 | 40.20±8.52 | 74.49±7.02 | 424.95±28.96 | 1358.00（1092.00，1641.50） |
|  | *t/Z* | -9.292 | 3.096 | 3.402 | -4.102 |
|  | *p* | ＜0.001 | 0.003 | 0.001 | 0.002 |
| T3 | 1 | 63.00±9.669 | 86.13±6.93 | 448.13±29.11 | 1822.00（1548.00，2115.00） |
|  | 0 | 39.98±8.577 | 80.03±6.49 | 428.65±27.30 | 1356.00（1139.00，1627.00） |
|  | *t/Z* | -11.267 | 4.087 | 3.105 | -4.370 |
|  | *p* | ＜0.001 | ＜0.001 | 0.003 | ＜0.001 |

Note: Group = 0 represents the control group; Group = 1 represents the intervention group.

Table 3. Model Effect Tests for MSES Scores

|  | *Wald X^2^* | DF | *P* |
| --- | --- | --- | --- |
| Group | 352.383 | 1 | <0.001 |
| Time | 210.919 | 2 | <0.001 |
| T0 | 191.004 | 1 | <0.001 |
| Time × Group | 197.249 | 2 | <0.001 |

Table 4. Comparison of the Main Effect of Time on MSES Scores

| Group | Parameter | *β* | Standard Error | 95%CI | *Wald X^2^* | *P* |
| --- | --- | --- | --- | --- | --- | --- |
| 0 | T3 | 0.068 | 0.150 | （-0.226,0.362） | 0.206 | 0.650 |
|  | T2 | 0.293 | 0.169 | （-0.039,0.625） | 2.998 | 0.083 |
|  | T1 | 0 |  |  |  |  |
| 1 | T3 | 11.225 | 0.896 | （9.469,12.981） | 156.891 | <0.001 |
|  | T2 | 8.875 | 0.737 | （7.431,10.319） | 145.127 | <0.001 |
|  | T1 | 0 |  |  |  |  |

Note: Group = 0 represents the control group; Group = 1 represents the intervention group.

Table 5. Comparison of the Main Effect of Group on MSES Scores

| **Variable** | *β* | Standard Error | 95%CI | *Wald X^2^* | *P* |
| --- | --- | --- | --- | --- | --- |
| T3* Group =1 | 23.907 | 1.153 | （21.646,26.167） | 429.802 | <0.001 |
| T3* Group =0 | 0 |  |  |  |  |
| T2* Group =1 | 26.533 | 1.645 | （18.910,23.753） | 298.097 | <0.001 |
| T2* Group =0 | 0 |  |  |  |  |
| T1* Group =1 | 15.853 | 1.360 | （10.718,14.751） | 153.162 | <0.001 |
| T1* Group =0 | 0 |  |  |  |  |

Note: Group = 0 represents the control group; Group = 1 represents the intervention group.

Table 6. Dimensional Scores of MSES in Both Groups at Week 12 (Mean ± SD)

|  | 1（n=40） | 0（n=41） | *t* | *p* |
| --- | --- | --- | --- | --- |
| Task Self-Efficacy | 24.53±4.78 | 16.98±4.97 | 6.969 | <0.001 |
| Coping Self-Efficacy | 13.60±3.71 | 9.34±2.88 | 5.785 | <0.001 |
| Scheduling Self-Efficacy | 23.68±4.88 | 16.83±4.16 | 6.805 | <0.001 |

Note: Group = 0 represents the control group; Group = 1 represents the intervention group.

Table 7. Model Effect Tests for 6MWD

|  | *Wald X^2^* | DF | *P* |
| --- | --- | --- | --- |
| Group | 70.630 | 1 | <0.001 |
| Time | 13.738 | 2 | 0.001 |
| T0 | 104.887 | 1 | <0.001 |
| Time × Group | 17.850 | 2 | <0.001 |

Table 8. Comparison of the 6MWD

| Group | Parameter | *β* | Standard Error | 95%CI | *Wald X^2^* | *P* |
| --- | --- | --- | --- | --- | --- | --- |
| 0 | T3 | 2.091 | 2.049 | （-1.926,6.107） | 1.041 | 0.308 |
|  | T2 | -1.690 | 1.664 | （-4.952,1.571） | 1.032 | 0.310 |
|  | T1 | 0 |  |  |  |  |
| 1 | T3 | 11.000 | 2.888 | （5.339,16.661） | 14.505 | <0.001 |
|  | T2 | 10.795 | 2.527 | （5.842,15.748） | 18.245 | <0.001 |
|  | T1 | 0 |  |  |  |  |

Note: Group = 0 represents the control group; Group = 1 represents the intervention group.

Table 9. Comparison of the Main Effect of Group on 6MWD

| **Variable** | *β* | Standard Error | 95%CI | *Wald X^2^* | *P* |
| --- | --- | --- | --- | --- | --- |
| T3* Group =1 | -29.168 | 3.748 | （-36.514,-21.822） | 60.568 | <0.001 |
| T3* Group =0 | 0 |  |  |  |  |
| T2* Group =1 | -32.725 | 3.447 | （-39.481,-25.968） | 90.124 | <0.001 |
| T2* Group =0 | 0 |  |  |  |  |
| T1* Group =1 | -20.239 | 3.870 | （-27.824,-12.654） | 27.353 | <0.001 |
| T1* Group =0 | 0 |  |  |  |  |

Note: Group = 0 represents the control group; Group = 1 represents the intervention group.

Table 10. Model Effect Tests for IPAQ

|  | *Wald X^2^* | DF | *P* |
| --- | --- | --- | --- |
| Group | 11.618 | 1 | 0.001 |
| Time | 7.529 | 2 | 0.023 |
| T0 | 13.650 | 1 | ＜0.001 |
| Time × Group | 1.789 | 2 | 0.409 |

Table 11. Comparison of Main Effects on IPAQ

| **Variable** | *β* | Standard Error | 95%CI | *Wald X^2^* | *P* |
| --- | --- | --- | --- | --- | --- |
| Group =1 | 498.624 | 146.3530 | （211.777，785.471） | 11.608 | 0.001 |
| Group =0 | 0 |  |  |  |  |
| T3 | 141.297 | 85.5866 | （-26.450，309.043） | 2.726 | 0.099 |
| T2 | 206.506 | 82.5966 | （44.620，368.392） | 6.251 | 0.012 |
| T1 | 0 |  |  |  |  |

Note: Group = 0 represents the control group; Group = 1 represents the intervention group.

Table 12. Model Effect Tests for Quality of Life

|  | *Wald X^2^* | DF | *P* |
| --- | --- | --- | --- |
| Group | 20.686 | 1 | ＜0.001 |
| Time | 689.115 | 2 | ＜0.001 |
| T0 | 120.811 | 1 | ＜0.001 |
| Time × Group | 3.255 | 2 | 0.196 |

Table 13. Comparison of Main Effects on CQQC

| **Variable** | *β* | Standard Error | 95%CI | *Wald X^2^* | *P* |
| --- | --- | --- | --- | --- | --- |
| Group =1 | -4.633 | 1.020 | （-6.632，-2.635） | 20.643 | ＜0.001 |
| Group =0 | 0 |  |  |  |  |
| T3 | 15.160 | 0.635 | （13.915，16.406） | 569.314 | ＜0.001 |
| T2 | 9.383 | 0.443 | （8.515，10.251） | 448.74 | ＜0.001 |
| T1 | 0 |  |  |  |  |

Note: Group = 0 represents the control group; Group = 1 represents the intervention group.
